# Quantitative 3D Mapping of the Human Skeletal Muscle Mitochondrial Network

**DOI:** 10.1016/j.celrep.2019.01.010

**Published:** 2019-01-15

**Authors:** Amy E. Vincent, Kathryn White, Tracey Davey, Jonathan Philips, R. Todd Ogden, Conor Lawless, Charlotte Warren, Matt G. Hall, Yi Shiau Ng, Gavin Falkous, Thomas Holden, David Deehan, Robert W. Taylor, Doug M. Turnbull, Martin Picard

**Affiliations:** 1 Wellcome Centre for Mitochondrial Research, Institute of Neuroscience, Newcastle University, Newcastle upon Tyne, UK; 2 MRC Centre for Ageing and Vitality, Newcastle University, Newcastle upon Tyne, UK; 3 EM Research Services, Newcastle University, Newcastle upon Tyne, UK; 4 Institute of Child Health, University College London, London, UK; 5 National Physical Laboratory, Teddington, UK; 6 Institute of Cellular Medicine, Newcastle University, Newcastle upon Tyne, UK; 7 Department of Biostatistics, Columbia University Mailman School of Public Health, New York, NY, USA; 8 Department of Psychiatry, Division of Behavioral Medicine, Columbia University Irving Medical Center, New York, NY, USA; 9 Department of Neurology and Columbia Translational Neuroscience Initiative, H. Houston Merritt Center, Columbia University Irving Medical Center, New York, NY, USA; 10 Columbia University Aging Center, Columbia University, New York, NY, USA; 11 Lead Contact

## Abstract

Genetic and biochemical defects of mitochondrial function are a major cause of human disease, but their link to mitochondrial morphology *in situ* has not been defined. Here, we develop a quantitative three-dimensional approach to map mitochondrial network organization in human muscle at electron microscopy resolution. We establish morphological differences between human and mouse and among patients with mitochondrial DNA (mtDNA) diseases compared to healthy controls. We also define the ultrastructure and prevalence of mitochondrial nanotunnels, which exist as either free-ended or connecting membrane protrusions across non-adjacent mitochondria. A multivariate model integrating mitochondrial volume, morphological complexity, and branching anisotropy computed across individual mitochondria and mitochondrial populations identifies increased proportion of simple mitochondria and nanotunnels as a discriminant signature of mitochondrial stress. Overall, these data define the nature of the mitochondrial network in human muscle, quantify human-mouse differences, and suggest potential morphological markers of mitochondrial dysfunction in human tissues.

## In Brief

Vincent et al. use 3D electron microscopy to provide a quantitative morphometric assessment of human skeletal muscle mitochondria. They find that healthy human muscle mitochondria differ from mouse mitochondria and show that primary mtDNA defects are associated with a distinct morphological signature including increased abundance of mitochondrial nanotunnels.

## Graphical Abstract

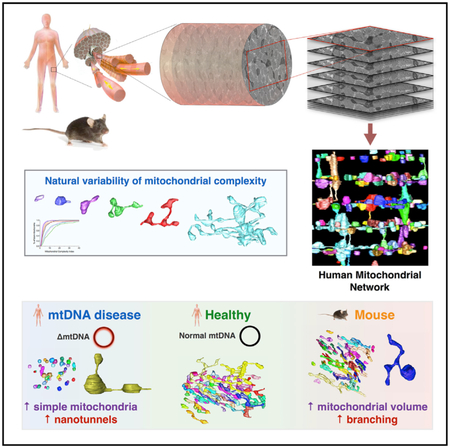

## INTRODUCTION

Mitochondria are multifunctional organelles that dynamically transition from punctate structures to branched elongated tubules within cells. Continuous changes in mitochondrial shape arise through fission and fusion of mitochondria. Importantly, a bidirectional relationship links mitochondrial shape and function (Liesa and Shirihai, 2013; Picard et al., 2013a). Changes in mitochondrial shape in isolated cellular systems occur within minutes to hours and precede signaling events in model systems, influencing skeletal muscle atrophy (Romanello et al., 2010), oxidative stress (Shenouda et al., 2011; Yu et al., 2008), metabolic sensing (Ramírez et al., 2017; Schneeberger et al., 2013), and lifespan (Weiret al., 2017). This underscores the biological significance of mitochondrial morphology transitions for cellular and organismal functions (Eisner et al., 2018) and emphasizes the need to visualize and quantify mitochondrial shapes to gain insight into the relevance of mitochondrial morphology for human health and disease.

One tissue that exhibits high energy consumption and contains a large number of mitochondria is skeletal muscle. Building on initial qualitative imaging revealing highly reticular mitochondrial networks in rodents (Bakeeva et al., 1978; Ogata and Yamasaki, 1997) and live cell imaging approaches to quantify mitochondrial morphology (Koopman et al., 2005), a quantitative method was developed allowing quantification of mitochondrial size and shape in two dimensions (Picard et al., 2013b). However, mitochondria exhibit complex three-dimensional and anisotropic arrangement (i.e., different morphology when measured in different orientation) in various cell types. This is particularly salient in skeletal muscle, as subsequently described in mice (Eisner et al., 2014; Glancy et al., 2015; Leduc-Gaudet et al., 2015; Picard et al., 2013b), but the morphological 3D characteristics of human mitochondria have not been described.

Consistent with their bacterial ancestry, mitochondria contain their own genome, the mitochondrial DNA (mtDNA), which encodes essential components of the respiratory chain and oxidative phosphorylation (OXPHOS) system required for ATP synthesis (Nicholls and Fergusson, 2013). Clinically, mtDNA mutations have been widely implicated in human disease and aging (Gorman et al., 2016; Nunnari and Suomalainen, 2012; Suomalainen and Battersby, 2018). In cellular and animal models, mtDNA mutations (Koopman et al., 2010; Picard et al., 2014), impaired Ca^2+^ handling (Eisner et al., 2017), and disrupted mitochondrial dynamics due to autosomal defects in the fusion-fission components (Ranieri et al., 2013) all perturb mitochondrial morphology. Furthermore, in human skeletal muscle fibers, mtDNA mutations that compromise mitochondrial energy production are also associated with ultrastructural abnormalities in mitochondrial cristae organization (Vincent et al., 2016), suggesting that mitochondrial morphology and function are also linked *in vivo*. However, the quantitative relationship between mtDNA defects, mitochondrial morphology, and mitochondrial organization in human disease remains unclear.

To clarify this relationship, we developed a quantitative approach to analyze 3D skeletal muscle mitochondrial network organization at single organelle resolution in human muscle biopsies. We provide quantitative analysis of mitochondrial morphology in human muscle, perform a comparative mouse-human analysis, and evaluate the effect of mtDNA mutations in patients with genetically confirmed primary mtDNA mutations on morphological parameters. We also use a combination of multivariate analyses to identify key mitochondrial morphology characteristics of the myopathic mitochondrial network, providing a foundation to examine and monitor anomalies in muscle mitochondrial networks.

## RESULTS

We imaged biopsy specimens from healthy volunteers (n = 8) by serial block face scanning electron microscopy (SBF-SEM). The resolution achieved here with SBF-SEM is 10 nm in the x/y plane and 30 nm in the z plane, which typically enables visualization of individual cristae membranes. This is inferior to conventional transmission EM imaging but superior to other three-dimensional light-based imaging methods (Dahl et al., 2015; Eisner et al., 2014; Picard et al., 2013b). Because our objective was to establish the morphology of individual mitochondria within the skeletal muscle network, we opted for a method with sufficient resolution to resolve closely juxtaposed (approximately ≥ 20 nm) membranes, which is critical to avoid merging immediately adjacent organelles with distinct outer mitochondrial membranes (Figure S1).

**Figure 1.**
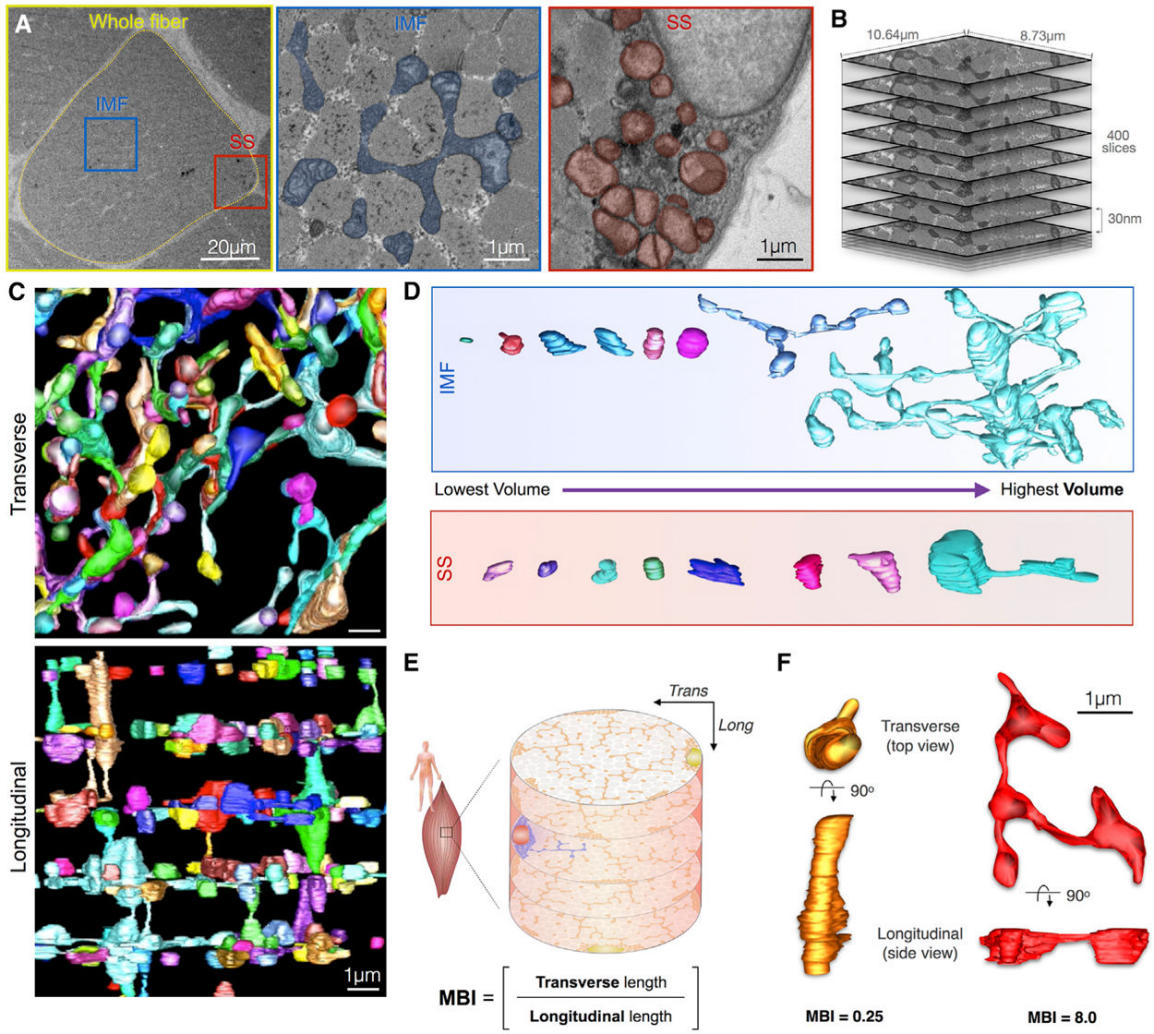
Imaging and Quantitative Analysis of Mitochondrial Morphology and Volume in IMF and SS Mitochondrial Subpopulations in Human Skeletal Muscle (A) Electron micrograph of human skeletal muscle in transverse (i.e., cross section) orientation. A single cell is highlighted (yellow), with corresponding higher magnification images of intermyofibrillar (IMF) and subsarcolemmal (SS) mitochondria. Note the difference in morphology between IMF and SS mitochondrial sub-populations. (B) Z stack at EM resolution from serial block face scanning electron microscopy (SBF-SEM) used for 3D reconstructions. See Video S1 for animation. Total imaging depth is 12 μm. (C) The human mitochondrial network was reconstructed and shown here in transverse (top view) and longitudinal (side view) orientations. Each mitochondrion is a different color. (D) The spectrum of human IMF and SS mitochondrial shapes and volumes, ranked left to right from smallest to largest. (E) Schematic of a skeletal muscle fiber and the different sarcomeric planes. The mitochondrial branching index (MBI) is used to quantify the relative branching in the transverse and longitudinal orientations. Trans, transverse; Long, longitudinal. (F) Two reconstructed mitochondria from SBF-SEM seen in both transverse and longitudinal orientations. The orange mitochondrion (left) is longer and more branched in the longitudinal orientation of the muscle fiber (i.e., columnar), whereas the red mitochondrion (right) is more extensively branched in the transverse orientation (i.e., in cross section). This branching anisotropy is captured by each mitochondrion’s MBI value.

To test our approach in human skeletal muscle, we first analyzed coexisting mitochondrial sub-populations known to markedly differ in both morphology and function (Ferreira et al., 2010; Picard et al., 2013b). The subsarcolemmal (SS) mitochondria reside beneath the plasma membrane, often in proximity to myonuclei (i.e., perinuclear) and capillaries (i.e., perivascular) (Glancy et al., 2014), and are generally globular in morphology, with few branches. On the other hand, the intermyofibrillar (IMF) mitochondria are located between myofibrils arranged in pairs at the z-band of each sarcomere (Vendelin et al., 2005) and have elongated tubular shapes when imaged in 2D along the transverse plane of the muscle in rodents (Figure 1A; Picard et al., 2013b).

For each region of interest, 400 serial images were captured at 30 nm intervals in the transverse orientation (Figure 1B; Video S1). Approximately 50 individual SS or IMF mitochondria were manually traced from each image stack and 3D reconstructions generated. In all human and mouse fibers analyzed, the mitochondrial network was found not to be a single reticulum. Rather, the mitochondrial network is composed of largely distinct organelles rarely reaching beyond a few microns in length in either direction, with the highest density of mitochondria at each of the sarcomeric planes (Video S1; Figure 1C). Reconstructions also confirmed that SS mitochondria tend to be globular and possess branches only where they project into the IMF space (Video S2), representing possible sites of physical interactions or their migration toward the IMF compartment.

### Establishing Quantitative Metrics of Three-Dimensional Shape Complexity and Branching Direction

To determine whether mitochondrial shapes significantly differ between cells, individuals, species, and in response to mtDNA defects, a quantitative measure of morphological complexity is required. We therefore adapted the two-dimensional metric known as form factor (FF) (Koopman et al., 2005) to a threedimensional metric of mitochondrial shape complexity. We call this the mitochondrial complexity index (MCI; see STAR Methods for details). The MCI is analogous to sphericity and scales with mitochondrial shape complexity, including branches and increased surface area relative to volume. Simulations on basic 3D models confirmed that MCI is insensitive to mitochondrial volume, such that mitochondria of the same shape but of different volumes have the same MCI (Figure S2).

Because mitochondria exchange molecular information with each other via fusion (Chen et al., 2010), a process that could influence the spread of mtDNA defects along mitochondrial branches in genetic mitochondrial disease, we also sought to develop a method to assess mitochondrial branching in either the transverse (i.e., across the muscle fiber) or longitudinal (i.e., along the fiber) orientation (Figures S3A and S3B). The ratio of quantified transverse/longitudinal branching yielded the mitochondrial branching index (MBI) (Figure 1E). Mitochondria with equal branching in both orientations have an MBI of 1; those with greater branching in the transverse plane score > 1, and those more branched along the length of the muscle fiber score < 1 (Figure 1F). Together, the MCI and MBI represent quantitative metrics to evaluate individual mitochondrial morphology in healthy humans, across species, and in disease.

### Biopsy to Fixation Delay Does Not Impact Mitochondrial Morphology

**Figure 2.**
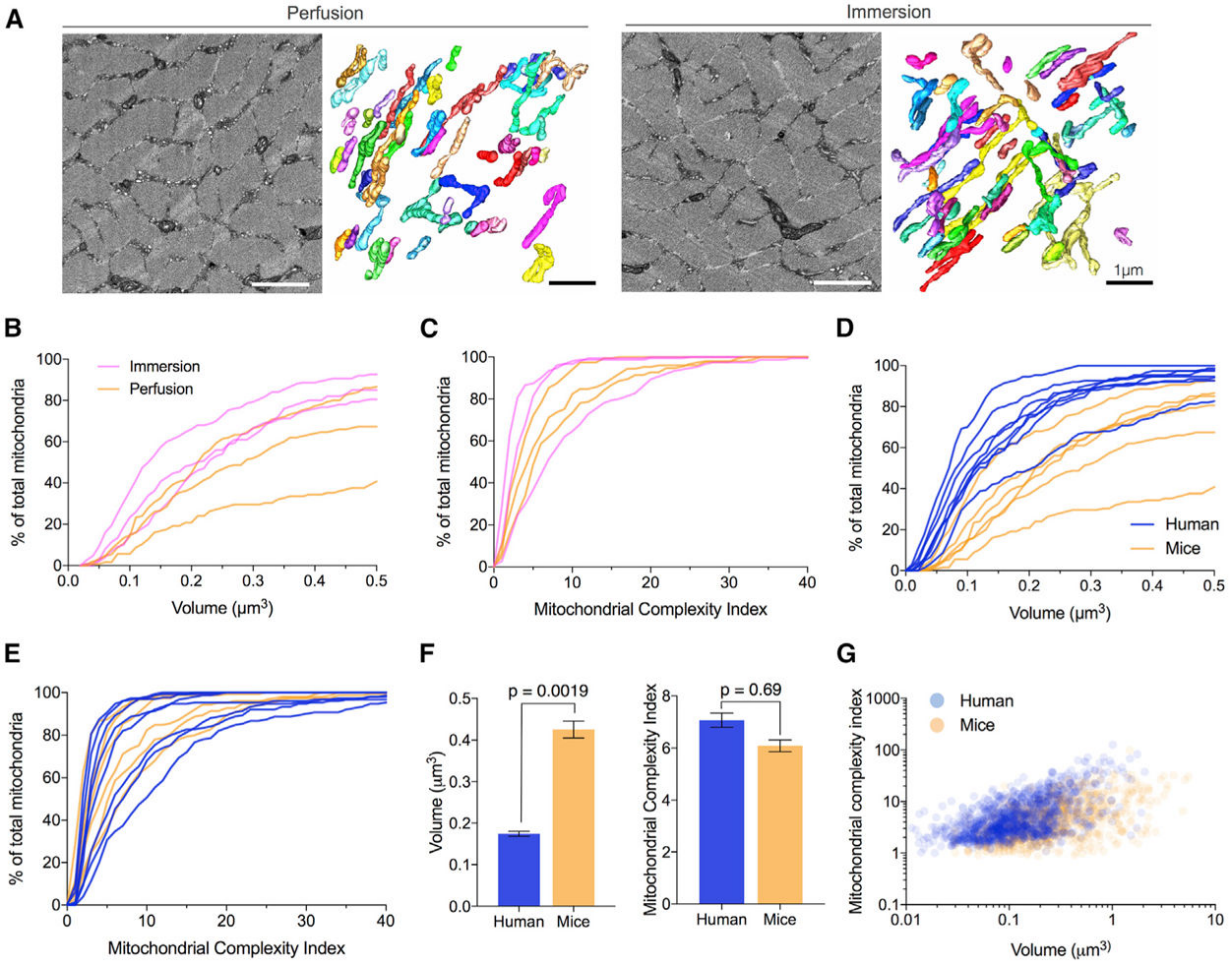
Mitochondrial Morphology Differs between Healthy Humans and Mice (A) Electron micrograph and 3D reconstruction of mitochondria in mouse tibialis anterior muscle. Muscle was fixed in two ways: by immersion after a delay at room temperature (left) and via transcradial perfusion without delay (right). Scale bars, 1 μm. (B and C) IMF mitochondrial volume (B) and MCI (C) in mouse muscle fixed by transcardial perfusion (orange) and immersion (pink) fixation shown as cumulative frequency distributions. Each line represents data from 150 mitochondria sampled across 3 muscle fibers in each animal. n = 3 mice per group. (D) Mitochondrial volume and (E) MCI in healthy humans (blue) and mice (orange) shown as cumulative frequency distributions. n = 6 mice and 8 humans. (F) Average MCI for human and mouse IMF mitochondria. Data are means ± SEM; n = 875 in mouse and 1,180 in humans, linear mixed model. (G) Bivariate plot of volume and MCI for healthy human controls (blue) and mice (orange). Each point represents a single mitochondrion.

Gaining the most clinically relevant insights into human cell biological processes requires the study of human tissues, but there are limitations associated with clinical studies. In live cultured cells and *ex vivo*, mitochondria undergo fusion events within minutes (Eisner et al., 2014) and morphology transitions occur in seconds to minutes (Liu et al., 2009). Therefore, it is conceivable that mitochondrial morphology would shift during the delay necessarily encountered between the biopsy of human muscle and subsequent fixation. To directly test this possibility, we compared muscle mitochondrial morphology when it was either (1) fixed *in vivo* without any delay via transcardial perfusion or (2) fixed by immersion following a 1 h post-mortem delay, in the mouse tibialis anterior (Figure 2). SBF-SEM image stacks were acquired as for human biopsies and high-resolution 3D models generated.

Three-dimensional reconstructions showed no significant difference between fixation methods (Figure 2A). Complex shaped mitochondria with branches >1 μm in length could be observed in equal abundance with both immediate and delayed fixation. Likewise, neither mitochondrial volume nor MCI values differed significantly across fixation conditions (Figures 2B and 2C), demonstrating that fixation delay does not result in loss of morphological complexity within an hour *ex vivo*. These results demonstrate that the mitochondrial network organization can be studied in fixed biopsy specimens.

### Mouse and Human Mitochondrial Morphology Differ

Qualitatively, the 3D human mitochondrial network (Figure 1C) appeared less interconnected than previous reports have demonstrated in mice (Glancy et al., 2015), but mitochondrial morphology had not previously been quantified and compared across mouse and human. We therefore quantified mitochondrial volume and MCI in parallel from both species. Mitochondrial populations were analyzed using cumulative frequency distributions that illustrate the relative abundance of mitochondria with different volume or MCI values (Figures 2D and 2E).

Mouse IMF mitochondria were 144.0% larger on average than in healthy humans (p = 0.0019) (Figure 2F) but did not differ on average in MCI (p = 0.69) (Figure 2G). In mice, the top 10% largest mitochondria had an average volume of 4.19 μm^3^, compared to only 0.68 μm^3^ for human mitochondria. Similarly, SS or perinuclear mitochondria were on average 412.0% larger (p < 0.0001) but also 31.0% less complex (p = 0.014) compared to those in healthy humans (Figure S4). Thus, mitochondrial volume, and to a lesser extent the complexity of SS mitochondria, differ significantly differ between mouse and human.

### Intermyofibrillar Mitochondria Are More Complex than Subsarcolemmal Mitochondria

**Figure 3.**
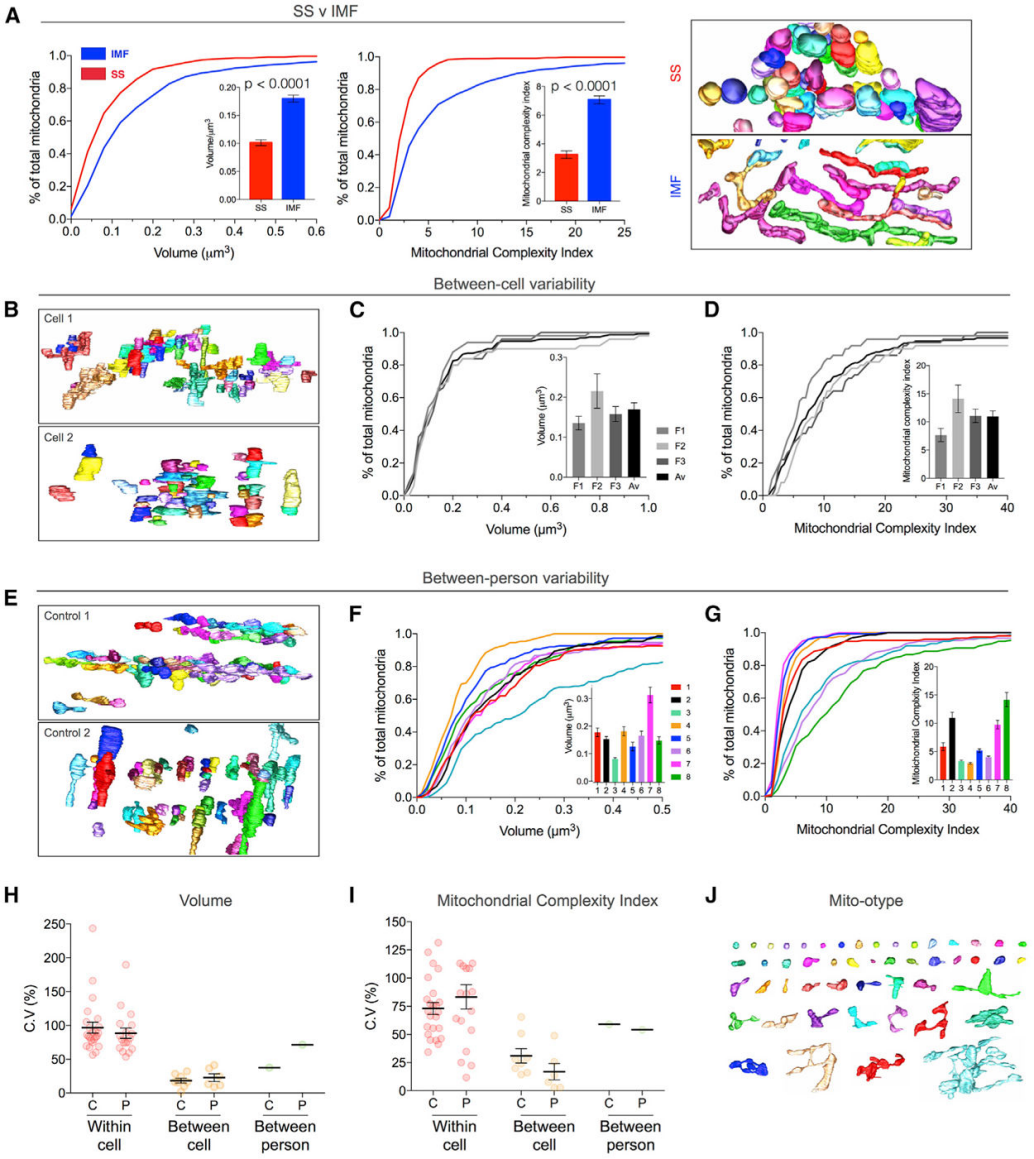
Natural Variation in Mitochondrial Volume and Complexity in Healthy Human Skeletal Muscle (A) Population distribution of mitochondrial volume and MCI in control human muscle shown as cumulative frequency distributions for SS and IMF mitochondria. Insets: mean values ± SEM; n = 346 for SS and 1,180 for IMF, linear mixed model. (Right) Representative 3D reconstructions of mitochondrial subpopulations. (B) Example 3D reconstructions of two muscle cells from the same person (Control 1). (C and D) Cumulative population distributions of mitochondrial volume (C) and MCI (D) in three muscle fibers from Control 1, and (inset) with means ± SEM for each cell. Each line represents 50 IMF mitochondria from a single muscle cell. (E) Example 3D reconstructions from muscle fibers from two different individuals (Controls 1 and 2) illustrating between-person (i.e., inter-individual) differences. (F and G) Cumulative frequency distribution demonstrating between-person variation in volume (F) and MCI (G) in 8 healthy individuals. Each line represents 150 IMF mitochondria sampled across 3 muscle fibers for each person. (H and I) Coefficient of variation (C.V.) for volume (H) and MCI (I) in healthy controls(*C*) and patients (*P*). C.V. values are shown between mitochondria within single cells (n = 18–24 cells), between the mean values across fibers (n = 6–8 individuals), and between-person across healthy control and mito disease groups(n = total of 1,180 and 900 IMF mitochondria in controls and patients, respectively). Data shown are mean ± SEM. (J) Karyotype-like arrangement of 50 individual reconstructed IMF mitochondria from a single muscle fiber of patient 5 (asymptomatic sister with m.8344A>G), from the lowest to the highest MCI (left to right). See Figure S4 for larger version and Video S3 for an animation of the source muscle fiber.

In healthy human muscle, volume for SS and IMF mitochondria were relatively similar. IMF mitochondria were on average 77.2% larger than SS mitochondria (p < 0.0001), largely due to a lower proportion of small IMF mitochondria (Figure 3A). In comparison, the MCI for human IMF mitochondria was 118.0% greater than for SS mitochondria (p < 0.0001), a difference largely attributable to a substantial proportion of highly complex IMF mitochondria, and a complete absence of SS mitochondria with MCI > 7.5 (Figure 3A).

The MCI population distribution also showed a higher level of kurtosis and skewness (kurtosis = 37.09, skewness = 4.88, n = 1201) for IMF mitochondria than for SS mitochondria (kurtosis = 27.14, skewness = 4.02, n = 344), possibly suggesting the existence of structural or other factors that constrain morphology, particularly in the IMF compartment.

SS mitochondria in patients with mitochondrial disease were on average 147.0% larger than those in healthy controls (p = 0.0076) but showed no difference on average in complexity (p = 0.50). Because the IMF compartment is the predominant compartment in muscle fibers, and given previous work showing that IMF are more responsive than SS mitochondria to metabolic perturbations such as exercise (Picard et al., 2013b), aging (Leduc-Gaudet et al., 2015), and metabolic oversupply (Picard et al., 2015a), we focused the remainder of our analyses on IMF mitochondria.

Next, we determined the degree of variation in IMF mitochondrial volume and MCI at different levels: (1) between mitochondria within single muscle fibers, (2) between fibers of a given individual, and (3) between different individuals (Figures 3B–3I). Variation in mitochondrial volume and MCI within muscle fibers of healthy controls was highest with an estimated coefficient of variation (C.V.) ranging from 56% to 243% for volume and from 34% to 131% for MCI. Standard deviation determined from linear multi-level models showed similar results for both volume (SD = 0.81) and MCI (SD = 0.68). These numbers illustrate the remarkable population heterogeneity and diversity of 3D mitochondrial volumes and shapes within human muscle fibers. The diversity of a cell’s IMF mitochondria is illustrated in a mito-otype (Figure 3J)—the mitochondrial equivalent to a karyotype of nuclear chromosomes (see Figure S5 for full size rendering).

Comparatively, differences in mean volume and MCI between muscle fibers of a given individual were more limited, with cell-to-cell C.V. ranging from 21% to 32% for volume and 14% to 65% for MCI (Figures 3B–3D). Again, standard deviation yielded a similar result for both volume (SD = 0.14) and MCI (SD = 0.26). Variability between healthy controls was surprisingly large and most pronounced for MCI (Figures 3E–3G). When combining all cells together and comparing the averages between individuals, the C.V. was 38% for volume (Figure 3H) and 59% for MCI (Figure 3I) and had a SD of 0.28 for volume and 0.43 for MCI. These data establish that considerable intra- and inter-individual differences exist in mitochondrial size and morphological complexity in humans, with variability in both volume and MCI being highest intracellularly (among mitochondria of a given cell), and lowest between cells of a given person.

Interestingly, the within-person variation in MCI was approximately two times greater than for volume (SD = 0.26 versus 0.14; Mann-Whitney, p < 0.0001). Moreover, the average MCI across muscle fibers of a given individual varied by as much as 65%, a phenomenon likely attributable to the mixed muscle fiber composition in human muscle (Gouspillou et al., 2014), which show different mitochondrial properties (Mishra et al., 2015). The greater variation in MCI than in volume also suggested that morphological complexity may be more sensitive to biochemical deficiency and inter-individual differences than volume. Therefore, we mainly focused our subsequent analyses on MCI.

### Patients with mtDNA Defects Have More “Simple” Fragmented Mitochondria than Healthy Controls

**Figure 4.**
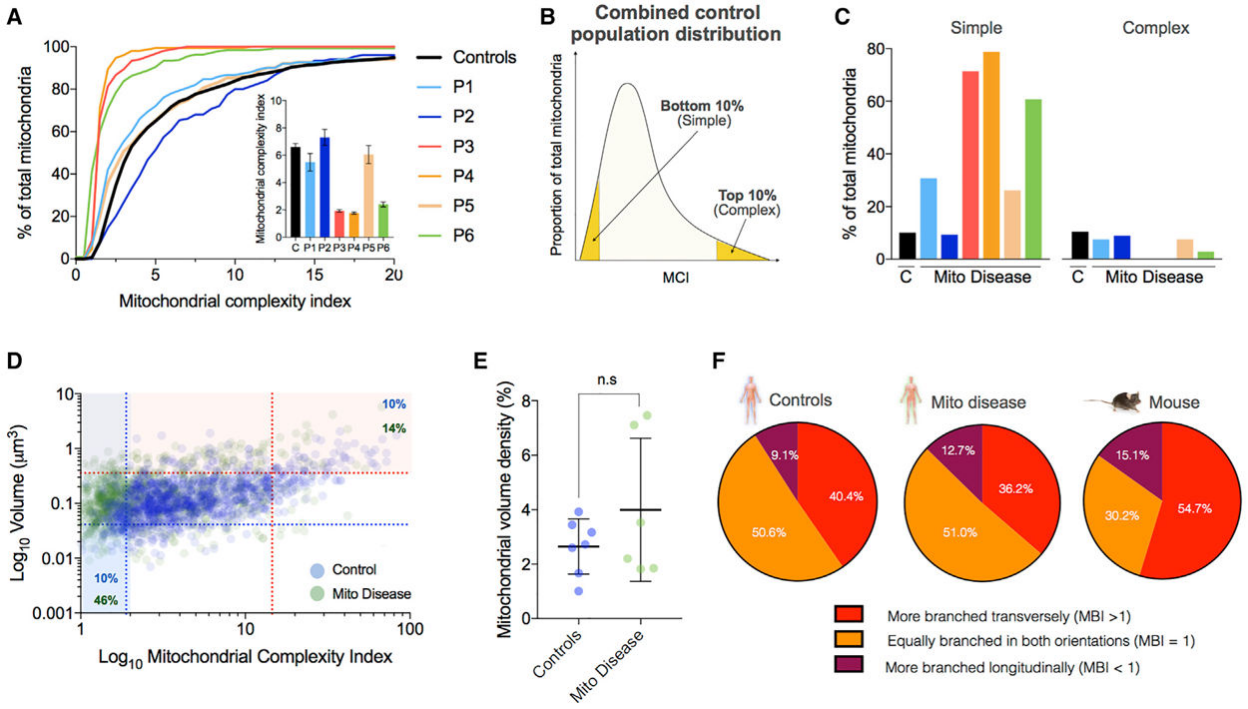
IMF Mitochondrial Volume and MCI in Patients with mtDNA Disease (A) Cumulative frequency distribution for MCI in combined healthy controls and patients. Each line in the patients represents 150 IMF mitochondria across 3 muscle fibers. Inset shows mean ± SEM for each person. Controls (n = 8) combined n = 1180 mitochondria. (B) Schematic illustrating “simple” and “complex” mitochondria defined as the 10^th^ and 90^th^ percentile of the control population distribution, respectively (shaded yellow regions). (C) Proportion of IMF mitochondria with MCI values that fall below the 10^th^ (simple) or above the 90^th^ (complex) percentiles of the control population for healthy controls (black) and patients 1–6 with mitochondrial disease. (D) Bivariate plot of MCI and volume for all healthy control IMF mitochondria (blue, n = 1,180) and mitochondrial disease (green, n = 900). Each data point represents a mitochondrion. Dotted lines denote the 10^th^ and 90^th^ percentiles of the control population. Simple mitochondria include 10% of mitochondria in the healthy controls and 46% in patients (blue shading). Large mitochondria include 10% of mitochondria in controls and 14% in patients (orange shading). (E) Mitochondrial volume density calculated as the proportion of muscle volume occupied by mitochondria. n = 8 controls and 6 patients, Mann-Whitney test, N.S. Data shown are mean ± SEM. (F) Mitochondrial branching index (MBI = Cross sectional ranching indicator/Longitudinal branching indicator) in combined controls (n = 973 IMF mitochondria), patients (n = 896) and mice (n = 839). Note that the number of mitochondria that are more branched cross sectionally (red) is greater than those more branched longitudinally (purple) in all three groups. Image stacks with imperfect orientation precluded reliable analyses of branching orientation for some mitochondria, which were excluded, explaining the lower total number of mitochondria quantified here.

We studied six patients with mtDNA disease including: single, large-scale mtDNA deletion (n = 2), m.8344A>G tRNA^Lys^ (n = 3), and m.3243A>G^Leu^(URR) (n = 1) (Table S1). These are among the most common disease-causing mtDNA defects (Gorman et al., 2015). As in healthy controls, MCI showed substantial intra- and inter-individual variability in patients with mitochondrial disease (Figure 4). The within fiber C.V. for MCI was 11%-170% (SD = 0.69), similar to controls (p = 0.72). In general, mitochondrial MCI and volume for patient mitochondria fall within the upper and lower limits of healthy controls, with a subset of fibers having a lower range in MCI values (Figure S6, e.g., patients 2 and 4).

Compared to healthy controls, mitochondria in patients were on average 39.6% less complex (p = 0.036) (Figure 4A). However, the MCI population distributions differed considerably in the abundance of “simple” (below the 10^th^ percentile of the control population) or “complex” (above the 90^th^ percentile of the control population) mitochondria. Mitochondria exist as functionally interconnected populations and only a portion of the overall population may respond to bioenergetic perturbations (Saunders et al., 2013). To capture these changes, we quantified the proportion of simple and complex mitochondria as shown in Figure 4B. Except for patient 2 who had a similar proportion of simple mitochondria as controls (9.33% versus 10%), 46% of mitochondria in patients with mtDNA defects were simple. This represents a 3.6-fold elevation in the proportion of simple mitochondria relative to healthy controls (Figures 4C and 4D).

### Mitochondrial Volume Density Does Not Account for Differences in MCI

MCI differences could theoretically be accounted for by variation in the overall density of mitochondria where more densely packed mitochondria may have more interconnections. We therefore measured mitochondrial volume density, defined as the percentage of muscle fiber volume occupied by mitochondria, in 3D reconstructed models over two full sarcomeres. Mitochondrial volume density was elevated in two patients but on average was not significantly different between patients (4.0%) and controls (2.6%) (p = 0.534) (Figure 4E). Across controls and patients, volume density was also not related to average MCI within a cell (Pearson r^2^ = 0.097, p = 0.19), which excluded the possibility that differences in MCI were driven by mitochondrial volume density. Taken together, these data from healthy controls and patients with mitochondrial disease demonstrate that mitochondrial dysfunction due to mtDNA mutations is associated with a shift in mitochondrial complexity independent of mitochondrial volume density.

### The Mitochondrial Network Is Anisotropic

MCI is agnostic to the orientation of individual mitochondria within the skeletal muscle fiber. However, clonally expanded mtDNA defects are thought to progress longitudinally along muscle fibers (Bua et al., 2006; Campbell et al., 2014) and we have recently reported that mtDNA dysfunction appears to preferentially spread in the transverse orientation prior to longitudinal propagation (Vincent et al., 2018). One potential mechanism for the cytoplasmic propagation of mtDNA mutations is the transmission of mtDNA molecules through fused mitochondria within the network. However, the relative connectivity of mitochondria in either transverse and longitudinal orientations, or mitochondrial “anisotropy,” has not previously been defined.

Mitochondria are organized at z-bands and occasionally extend in the longitudinal orientation to span a full sarcomere (see Video S3). In healthy controls, 3.3% of mitochodnria spanned a full sarcomere, compared to 4.9% (p = 0.07) in patients with mitochondrial disease. In contrast, mouse skeletal muscle contains a significantly larger fraction of columnar mitochondria that extend in the longitudinal axis, resulting in 18.9% of organelles spanning at least one sarcomere.

To precisely quantify the degree of mitochondrial connectivity in different orientations, we computed the MBI (see Figure 1E) for all IMF mitochondria. In healthy controls, approximately half (50.6%) of mitochondria were equally branched in both directions (Figure 4F). However, there were ∼4.4 times more mitochondria that are more extensively branched in the transverse orientation (40.4%) compared to the longitudinal orientation (9.1%) (Figure 4F). In mitochondrial disease, a similar percentage (51.0%) mitochondria were equally branched in both directions and a smaller fraction (36.2%) exhibited more branching in the transverse orientation (Figure 4F). Together, these data show that mitochondrial network connectivity across the plane of each z-band is approximately 4-fold greater than connectivity along the muscle fibers (see complete 3D reconstructions in Video S3) and that mtDNA defects may promote transverse mitochondrial branching within individual sarcomeric planes.

We next determined whether branching direction differed between humans and mice. Compared to healthy humans, mice had a smaller (30.2%) proportion of mitochondria equally branched in both orientations, and more mitochondria with extensive branching in both the transverse (54.7%) and longitudinal (15.1%) orientations. Mice have significantly more mitochondria with predominant branching in transverse (54.7% versus 40.4%, 1.35-fold of controls) and longitudinal (15.1% versus 9.1%, 1.66-fold) orientations than human mitochondria (Figure 4F). These quantitative data demonstrate that mouse skeletal muscle harbors a substantially different mitochondrial connectivity pattern than human muscle.

### High Mutation Load Is Associated with Mitochondrial Fragmentation

**Figure 5.**
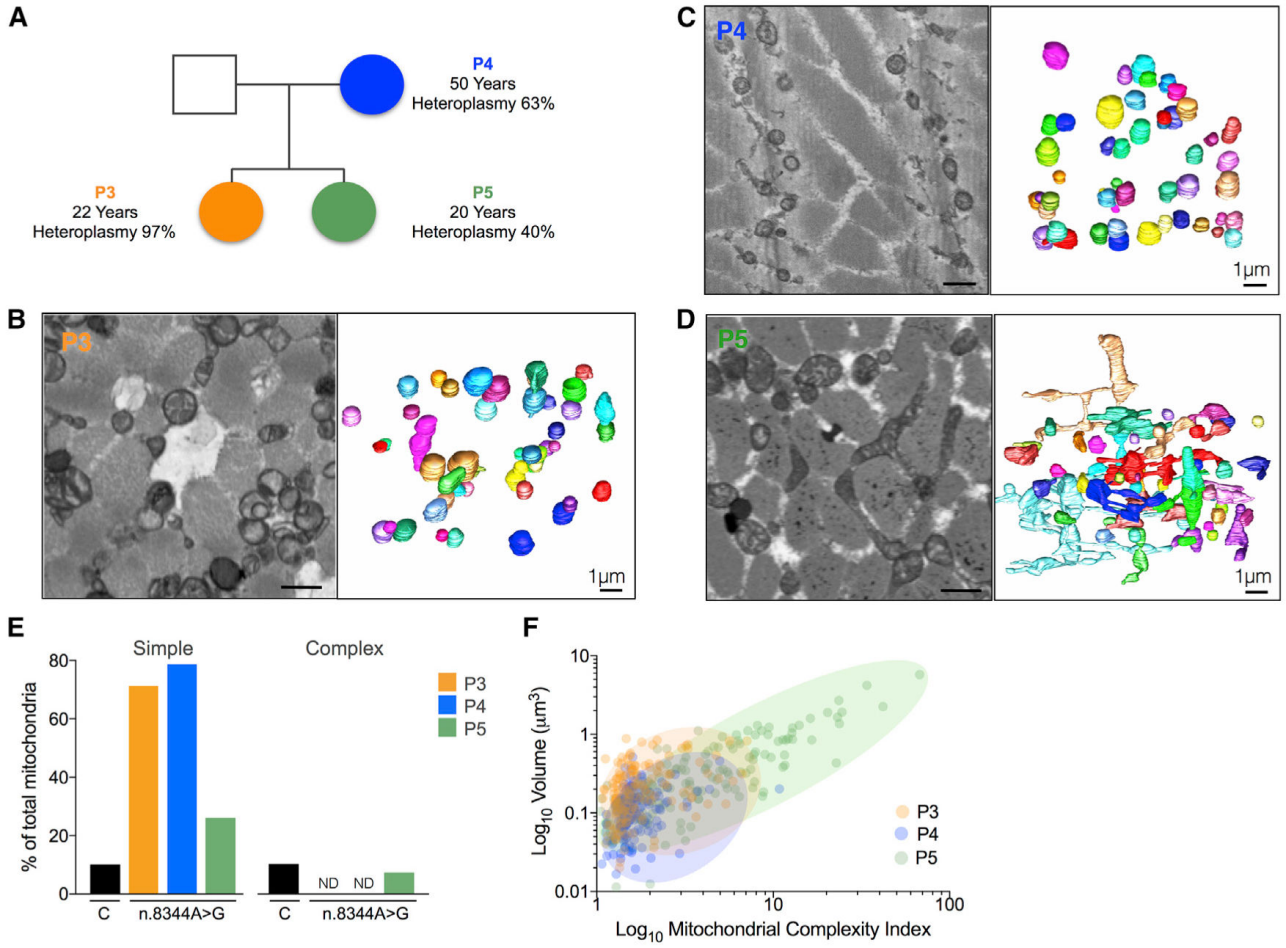
Variability in Mitochondrial Morphology in Three Family Members with Variable Levels of the m.8344A>G Mutation (A) Pedigree of three related female patients. Patient 3 (orange): the proband with 97% heteroplasmy, 97% COX-deficient muscle fibers, and severe myopathy and exercise intolerance; patient 4 (blue): the affected mother with 63% heteroplasmy, 22% COX-deficient muscle fibers, and mild myopathy; and patient 5 (green): the unaffected sister with 40% heteroplasmy and some intermediate COX-deficient fibers. The sisters are of comparable age but have inherited different levels of mtDNA mutation. (B-D) Single representative section from SBF-SEM and reconstruction of 50 IMF mitochondria in muscle fibers for of patients 4 (B), 3 (C), and 5 (D). Note the small fragmented morphology in the proband and mother (B and C) and a high number of branched mitochondria in the unaffected sister, who has intermediate mtDNA mutation load (D). Scale bars, 1 μm. (E) Proportion of simple and complex IMF mitochondria (based on the 10^th^ and 90^th^ percentiles of the MCI distributions in healthy controls) for patients 3, 4, and 5 compared to the healthy individuals. ND, no detectable complex mitochondria. (F) Bivariate plot of MCI and mitochondrial volume in the three individuals with the m.8344A>G mutation. Shaded areas illustrate the mitochondrial population distribution for each patient. Each data point is a mitochondrion (n = 150 per individual).

Within the group of patients with mitochondrial disease studied here, we had the opportunity to study samples from a trio of genetically related patients with the m.8344A>G tRNA^Lys^ mutation (Figure 5A, Videos S4, S5, and S6). The mother (patient 4) was 50 years old with 63% mutation load and showed a mild myopathy. Her eldest daughter (patient 3) was 22 years old, with skeletal muscle mutation load of 97%, severe myopathy, and exercise intolerance. In comparison, her younger daughter (patient 5) was 20 years old with only 40% mutation load in the muscle and was clinically asymptomatic. Given the genetic relatedness of these individuals, the similar age of the sisters, and the markedly different mtDNA heteroplasmy between them, this provided an opportunity to evaluate the association between mtDNA mutation load and mitochondrial morphology.

The 3D reconstructions for all three patients showed dramatic differences associated with heteroplasmy. Both the mother (patient 4) and the most severely affected daughter (patient 3) had the highest level of “simple” fragmented rounded mitochondria in this cohort: 71.3% and 78.7% of all mitochondria, respectively, compared to 10% in controls (Figures 5B and 5C, Videos S4 and S5). This is in sharp contrast to mitochondrial network architecture in healthy controls and in the unaffected sister (patient 5; Figure 5D and Video S6), the latter showing a large number of complex mitochondria.

The unaffected sister (patient 5, 40% heteroplasmy) was the only member of the family to have any complex mitochondria (7.3%), many of which had very high MCI values reflecting extensive branching and nanotunnels (Figure 5E). This patient also had 2.6 times more small mitochondria than controls, resulting in substantial heterogeneity of both MCI and volume (Figure 5F) associated with this intermediate level of mtDNA heteroplasmy.

### Mitochondrial Nanotunnels Are More Frequent in Patients than Controls

**Figure 6.**
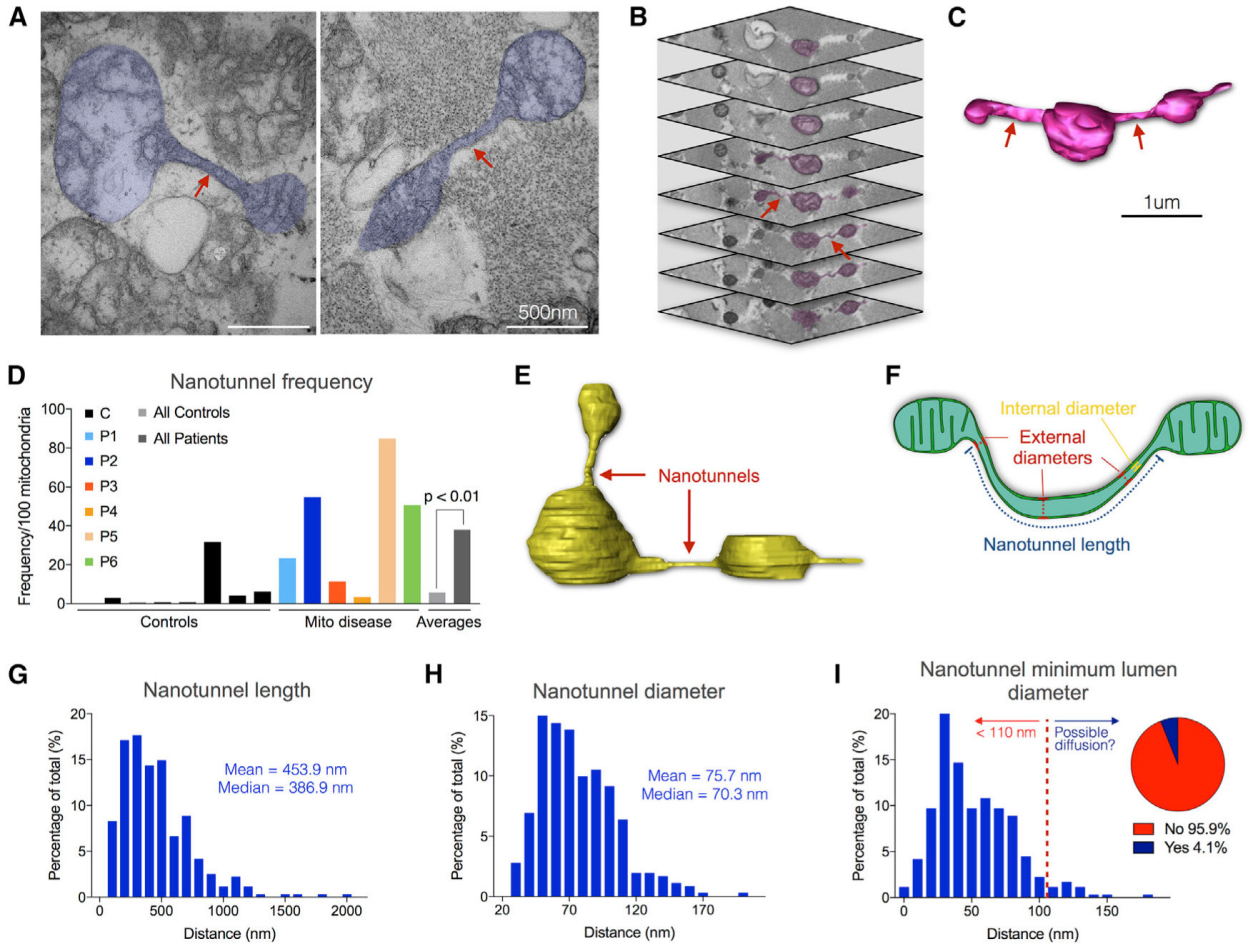
Prevalence and Anatomy of Mitochondrial Nanotunnels in Human Skeletal Muscle (A) Transmission Electron Microscopy of skeletal muscle from patient 5, with mitochondria harboring nanotunnels (pseudocolored blue). Arrows indicate nanotunnel shafts. (B and C) Image stack from SBF-SEM (B) and three-dimensional reconstruction (C) of a mitochondrion with two nanotunnels (arrows). Slice thickness is 30 nm. (D) Frequency of nanotunnels per 100 mitochondria in healthy controls and individuals with mtDNA disease. All controls (n = 8) were compared to all patients (n = 6), n = 399 nanotunnels, Mann-Whitney. (E and F) A mitochondrion with two nanotunnels reconstructed in Microscopy Image Browser (E) and schematic illustrating the measurements of nanotunnel anatomy obtained from 3D reconstructions (F). The internal diameter is derived from the external diameter (see I). (G and H) Frequency distribution of nanotunnel length (G) and diameter (H) as assessed from reconstructions measured in Amira (n = 362). Distributions are positively skewed. (I) Frequency distribution of minimum estimated lumen diameter for each nanotunnel measured by subtracting distances for the combined thickness of the intermembrane space, OMM, and IMM from the measured external diameter. The red dotted line indicates the estimated dimension of mtDNA nucleoids (based on bovine heart high-resolution imaging; Kukat et al., 2011). Inset: proportion of mitochondrial nanotunnels whose lumens are >110 nm, indicating the theoretical proportion of nanotunnels that could potentially house or transport nucleoids (right of red dashed line). Estimates based on n = 362 nanotunnels.

Mitochondrial nanotunnels are thin double membrane projections of both outer (OMM) and inner (IMM) mitochondrial membranes connecting two non-adjacent mitochondria and capable of transporting proteins between connected organelles (Figures 6A–6C; Eisner et al., 2014, 2017; Vincent et al., 2017). There is no previous systematic analysis of nanotunnel size and frequency in human tissues.

Using SBF-SEM, we identified nanotunnels in both the transverse and longitudinal orientation. In healthy controls, there was on average 2.1 nanotunnels per 100 mitochondria (Figure 6D). In comparison, patients with mitochondrial disease showed an average of 38 nanotunnels per 100 mitochondria, representing an 18.1-fold greater frequency (p < 0.01) (Figure 6D). It should be noted that patients 3 and 4 (the mother and eldest daughter with m.8344A>G mutation), despite exhibiting a massively fragmented mitochondrial network, still had an equal or greater nanotunnel frequency than most controls.

Mitochondrial nanotunnels can exist in two forms: as free-ended membrane protrusions arising from a donor mitochondrion or as connecting nanotunnels joining two mitochondria (Vincent et al., 2017). Of the 399 nanotunnels analyzed in mtDNA disease biopsies, 19.0% were free-ended membrane protrusions whereas 81.0% were connected to mitochondria at both ends.

We then determined their dimensions with measurements performed in Amira from high-resolution 3D reconstructions generated in microscopy image browser (MIB) (Figures 6E and 6F). Previous estimates of human mitochondrial nanotunnel dimensions were solely based on 2D single-plane transmission EM images (Vincent et al., 2017). Here, human skeletal muscle nanotunnels ranged from 64.9 nm to 2.0 μm in length (Figure 6G). The external diameters ranged from 26.1 to 204.2 nm (Figure 6H), dimensions similar to mitochondria-derived vesicles (Cadete et al., 2016; Soubannier et al., 2012).

To determine whether mitochondrial nanotunnels could theoretically transport mutant mitochondrial nucleoids or exhibit cargo selectivity, we estimated the smallest nanotunnel matrix lumen, which would theoretically limit the diffusion of macromolecules. We measured the smallest external diameter for each nanotunnel and subtracted 24 nm (8 nm for each the intermembrane space, plus 2 nm for OMM and IMM, multiplied by 2 sides). The distribution of estimated nanotunnel lumen size ranged from 2.1 to 180.2 nm (Figure 6I)—almost two orders of magnitude. Given that nucleoids are believed to be approximately 110 nm in size (Kukat et al., 2011), this limited structural evidence suggests that only 4.1% of nanotunnels measured in human muscle would be wide enough to allow the transport of mtDNA packaged as nucleoids. This point is in definite need of empirical validation.

### Key Mitochondrial Morphological Features Can Distinguish Patients from Controls

Finally, we sought to determine whether there are consistent mitochondrial morphological features that differentiate healthy controls from patients with mitochondrial disease. To test this hypothesis in an unbiased manner, we assembled a list of morphological features extracted from single mitochondria and population distributions, including the percentage of simple, complex, small, and large mitochondria (as described above), median MCI and volume, mitochondrial volume density, and mitochondrial nanotunnel frequency.

**Figure 7.**
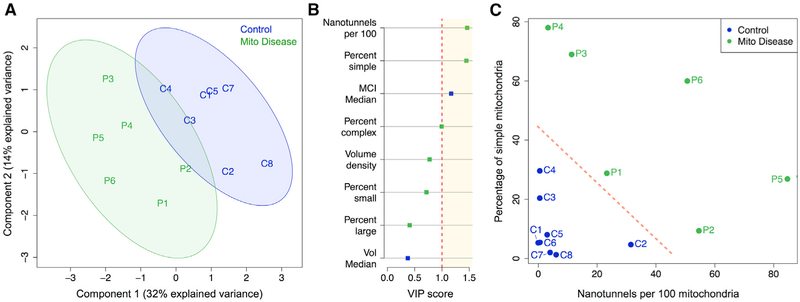
Multivariate Analysis Mitochondrial Morphological Signatures among mtDNA Disease and Healthy Controls (A) Partial least-squares discriminant analysis (PLS-DA) on mtDNA disease patients (n = 6) and healthy controls (n = 8) with 95% confidence intervals (shaded areas). The 2-component model illustrated explains 46% of the variance in the dataset and produces partial group separation. (B) Variable importance in projection (VIP) scores illustrating the contribution of different morphological features to the separation of healthy controls and patients in the PLS-DA model. Parameters with scores above 1 (red dotted line) are considered significant. Morphological features with higher values in mitochondrial disease are in green, those higher in healthy controls are in blue. Support vector machine (SVM) yielded similar results (not shown). (C) Bi-variate plot of the top two parameters, nanotunnels per 100 mitochondria and the proportion of simple mitochondria. Collectively, in this limited sample, this combination of measures represents a signature sufficient to accurately distinguish healthy controls (blue) and patients with mtDNA disease (green).

We used partial least-squares discriminant analysis (PLS-DA) to integrate morphological parameters into a single multivariate model that aims to maximize group differences (healthy controls versus mitochondrial disease). The model captured 46% of the total variance in the dataset and achieved partial separation of controls and patients with a small degree of overlap in the 95% confidence intervals (Figure 7A). Rank ordering of the variable importance in projection (VIP) scores for each parameter identified the most important morphological features that discriminate between healthy controls and mitochondrial disease. The top two features were (1) the abundance of nanotunnels and (2) the proportion of simple mitochondria (Figure 7B). As a validation of this result, the same two features were identified using another machine learning-based classifier, support vector machine (SVM), which similarly classifies data into two groups and provides estimates for the importance for each variable in the model (Ben-Hur et al., 2008). Plotting together the percentage of simple mitochondria against the abundance of nanotunnels resulted in complete segregation of healthy controls and patients (Figure 7C). These analyses, albeit underpowered, suggest that a high proportion of morphologically simple mitochondria combined with numerous nanotunnels may represent a signature of mitochondrial OXPHOS deficiency or mitochondrial stress.

## DISCUSSION

The morphology of mitochondria is intrinsically linked to their function, with numerous studies finding correlations between metabolic perturbations, mitochondrial morphology, mitochondrial function, and tissue functions in model organisms (Gomes et al., 2011; Hara et al., 2014; Leduc-Gaudet et al., 2015; Picard et al., 2014; Trushina et al., 2012). Here we developed threedimensional shape metric, the MCI, and validated it against known differences between skeletal muscle SS and IMF mitochondria. Using a combination of imaging and computational methods, we have provided quantitative three-dimensional analysis of human mitochondria and established differences in muscle mitochondrial morphology between human and mouse and between healthy controls and patients with primary mtDNA disease.

Previous three-dimensional reconstruction of human skeletal muscle mitochondria had been limited to one study using focused ion beam (FIB)-SEM in healthy individuals (Dahl et al., 2015), which did not quantify individual mitochondria. Another study using FIB-SEM also provided an assessment of mouse skeletal muscle mitochondrial morphology suggesting the existence of a highly connected mitochondrial reticulum (Glancy et al., 2015). Our analyses extend these findings in several ways.

Quantitative assessment of individual mitochondrial morphology in healthy human muscle fibers demonstrated an unexpectedly large degree of variability within each cell, between cells of the same individual, and between individuals. As expected, given that muscle fiber types with different oxidative capacity exhibit different mitochondrial morphology and dynamics (Dahl et al., 2015; Mishra et al., 2015), the variation in MCI between muscle fibers and between individuals was substantial. It is not currently possible to assess muscle fiber types on electron microscopy samples. Here, muscle fibers were selected based on high mitochondrial mass, so the analyses likely include a mixture of different oxidative fiber types. In patients with mitochondrial disease, cell-to-cell heterogeneity could be further enhanced by the heterogenous distribution of mtDNA mutation load presenting as a mosaic pattern of affected (OXPHOS-deficient) and unaffected (OXPHOS-normal) fibers (Rocha et al., 2015). Using the present technique, the correlation with single-cell heteroplasmy, respiratory chain function, and fiber type is not possible and remains a challenge for future studies to address. Nevertheless, even in analyses of single muscle fibers, IMF mitochondrial volume, complexity, and branching showed high heterogeneity—with simple and complex, and small and large mitochondria coexisting within the same cells. It is noteworthy that moderate mtDNA heteroplasmy in mitochondrial disease was associated with greater heterogeneity, as expected from having some mitochondria with mutant mtDNA and some with wild-type (i.e., normal) mtDNA. Together, these data document the natural heterogeneity of the human mitochondrial network, which by itself may represent a relevant outcome measure and may have functional implications in health and disease.

When comparing mouse and human muscle fibers, our data show that species differences are more pronounced for volume than for morphological complexity. Mitochondria in mice tend to be columnar, extending in the longitudinal orientation, with frequent small transverse protrusions similar to that reported previously (Glancy et al., 2015). Glancy et al. (2015) reported the mitochondrial network in oxidative mouse muscle fibers to constitute an almost completely continuous reticulum. In contrast, our analyses in mouse muscle reveal numerous distinct mitochondria are tightly packed into columns between myofibrils. These mitochondria frequently have immediately adjacent outer mitochondrial membranes and inter-mitochondrial junctions (IMJs) (Picard et al., 2015b). When visualized with sufficient resolution, these juxtaposed mitochondria are physically distinct organelles with separate matrix spaces (see Figure S1). Molecular information is certainly transferred between mitochondria under specific cellular states (Glancy et al., 2017; Kurz et al., 2010; Picard et al., 2015b). Nevertheless, rather than establishing continuity by proximity, in an effort to map the morphology of individual mitochondria, here we established segmentation criteria where each mitochondrion is defined as having a continuous outer membrane. The current approach reflects the anatomy and nature of individual mitochondria as ultimately determined by processes of fusion and fission. Moreover, immediately adjacent mitochondria can exhibit localized and isolated depolarization and “flash” events (Hou et al., 2014), establishing individual adjacent mitochondria as functional units under normal conditions. Thus, future morphology studies aiming to understand the reshaping of mitochondria under conditions of stress and disease should aim to combine both assessments of physical proximity and communication (Glancy et al., 2017; Picard et al., 2015b), and importantly, to develop quantitative morphological maps of mitochondrial networks involving the segmentation of individual mitochondria. A paper using a similar approach was published while the present article was under review (Bleck etal., 2018).

An unresolved question in mitochondrial biology is the relation between respiratory chain dysfunction, mitochondrial elongation, and fragmentation. Based on in vitro studies, the mitochondrial network has been demonstrated to undergo stress-induced mitochondrial fusion in response to low levels of stress or mutational heteroplasmy (Leduc-Gaudet et al., 2015; Picard et al., 2014; Shutt and McBride, 2013; Tondera et al., 2009). However, when mutational heteroplasmy is increased or stress exceeds some functional threshold, the mitochondrial network undergoes fragmentation. Metabolic challenge, such as starvation, induces a similar initial pro-fusion response, followed by fragmentation (Gomes et al., 2011; Rambold et al., 2011). Our data in the m.8344A>G tRNA^Lys^ trio lends some support for the validity of this model *in vivo*. Both the mother and her affected daughter with 60% and 97% mutation load, respectively, have fragmented mitochondrial networks. In comparison, the sister with 40% heteroplasmy of the same mutation in fact shows the highest mitochondrial network connectivity and number of nanotunnels of all studied individuals, including controls, consistent with a compensatory hyperfusion response to low levels of stress.

The current study also demonstrates that the mitochondrial network is anisotropic. Mitochondrial branching in humans is approximately four times more extensive across the transverse z-band than longitudinally. Not only does this reinforce the notion that three-dimensional assessments of mitochondrial morphology are more accurate than two-dimensional ones in muscle samples, but it also has implications for how mtDNA defects spread through muscle fibers. It has long been recognized that mitochondrial dysfunction is segmental along muscle fibers (Bua et al., 2006; Elson et al., 2002; Matsuoka et al., 1992) and expands over time (Lopez et al., 2000). However, an understanding of how the spread of mtDNA molecules is spatially constrained has remained elusive because it is not currently possible to track this process in real time. If mtDNA defects spread more rapidly along continuous mitochondrial tubules (i.e., the path of least resistance), our results demonstrating the anisotropic nature of the mitochondrial network—with four time more branching transversely than longitudinally—would predict that mitochondrial dysfunction (mtDNA mutations and respiratory chain deficiency) is likely to propagate more rapidly in the transverse orientation, rather than longitudinally. Both this finding and the recent identification of early segments of respiratory chain deficiency that are shorter longitudinally than the diameter of the muscle fiber (Vincent et al., 2018) converge to suggest that human skeletal muscle mtDNA mutations preferentially spread after following the natural connectivity of the mitochondrial network. However, given the lack of kinetic and experimental data, these observations should be interpreted with caution. In mice, which exhibit more longitudinal columnar mitochondria and a different and more extensive connectivity pattern than human mitochondria, the distribution of mtDNA defects and their propagation in disease could follow different kinetics and be driven by different mechanisms.

In an effort to identify predictive patterns in our data that would distinguish healthy individuals from those with mtDNA defects, we used an integrative machine learning approach. Contrary to conventional statistical tests that draw inference about a given sample, machine learning classifiers identify generalizable predictive patterns from the data (Bzdok et al., 2018). These approaches are data driven (i.e., do not require prior knowledge about which measures are more important) and handle datasets that have a large number of variables relative to the sample size (Pedro, 2012), such as in this study. We integrated 3D EM data in multivariate classifiers and discovered that a high prevalence of simple mitochondria and a high nanotunnel density may be discriminant morphological features of mitochondrial disease patients. It could be that these morphological features are specific to mitochondrial disease, or instead that they represent more generic morphological changes to energetic stress. A systematic comparison with non-mitochondrial neuromuscular disorders would be necessary to establish to what extent this signature is both sensitive and specific to mtDNA mutations.

Nanotunnels are bacteria-like thin double OMM-IMM membrane connections between mitochondria, capable of transporting proteins between physically constrained mitochondria (Vincent et al., 2017). Although the link between mtDNA mutations and increased mitochondrial nanotunelling is unclear, similar to compensatory hyperfusion as an initial stress response, we speculate that nanotunnels arise from mitochondria under stress that “reach out for help.” In this scenario, mitochondrial nanotunnels would enable functional complementation through the diffusion of proteins, ions, transcripts, and possibly membrane potential between physically constrained mitochondria that are not close enough to undergo fusion. Many questions remain unanswered around mitochondrial nanotunnels including whether their lumen is wide enough to transport mtDNA packaged as a proteinaceous nucleoids. Our three-dimensional measurements indicate that only a small fraction (4.1%) of nanotunnels would be wide enough to accommodate mtDNA nucleoids (based on bovine cryo-EM measurements). Yet, their diffusion or transport would likely require additional space to enable motility and directional motion within the nanotunnels. This remains an unresolved point in need of further investigation.

In conclusion, this work presents a quantitative EM resolution three-dimensional assessment of mitochondrial morphology and network organization in human skeletal muscle. This study also identifies morphological features that distinguish human from mouse skeletal muscle mitochondria, and healthy individuals from patients with mtDNA mutations, including an elevated frequency of simple mitochondria and mitochondrial nanotunnels in mitochondrial disease. The existence of mitochondrial nanotunnels suggests a possible mechanism for mitochondrial communication in human tissues, and a possible disease marker. Quantitatively defining the 3D morphological and anisotropic characteristics of mitochondrial networks in different human tissues should yield further insights into the mechanism underlying the origin and propagation of mitochondrial dysfunction in aging and disease.

## STAR★METHODS

### CONTACT FOR REAGENT AND RESOURCE SHARING

Further information and requests for resources and reagents should be directed to and will be fulfilled by the Lead Contact, Martin Picard (martin.picard@columbia.edu).

### EXPERIMENTAL MODELS AND SUBJECT DETAILS

#### Human subjects and patients

This study was approved by the Newcastle and North Tyneside Local Research Ethics Committees (reference 2002/205) and prior informed consent was obtained from each participant. All experiments were carried out in accordance with the approved guidelines. Biopsies were obtained from six patients with genetically-confirmed mtDNA disease; two patients with single, large mtDNA deletions, three patients with the m.8344A>G mutation and one with the m.3243A>G mutation (Table S1). In all cases, muscle biopsies were obtained from the tibialis anterior muscle. A further eight control biopsies from the distal part of the hamstring were collected from adults undergoing anterior cruciate ligament surgery (Table S2). For both patients and controls a section of the biopsy specimen was processed for EM, including gentle teasing of small muscle fiber bundles to insure rapid fixation.

#### Mouse muscle samples

All animal experiments were conducted in compliance with the UK Home Office (PPL 60/4455) and the Newcastle University Animal Welfare Ethical Review Board. Six male PV-Cre Knockin mice on c57bl/6N background (Jackson lab 008069) aged 5 (n = 2) and 7 (n = 4) months were used for experiments. Pairs of age-matched mice were processed in parallel, one underwent transcardial perfusion and one had a lethal injection of 200 μl of euthatal at 20 mg/mL containing sodium pentobarbital. The transcardially perfused mice were perfused with 50 mL of EM fixative (2% glutaraldehyde in 0.1M cacodylate buffer), during fixation the hind limb was attached to a spatula at a right angle preventing unwanted muscle contraction. All mice were dissected and the right tibialis anterior muscle carefully removed. The muscle was dissected further to remove a small region, the muscle fiber bundles teased apart and immersed into fixative.

### METHOD DETAILS

#### Transmission electron microscopy

For all samples, muscle fiber bundles fixed overnight in 2% glutaradehyde (in cacodylate buffer (0.1M, pH7.4)) at 4°C. Fibers were post fixed in 1% osmium tetroxide for 1 hour. Samples were dehydrated in a graded series of acetone (25%, 30 min; 50%, 30 min; 75%, 30 min; 2×100%, 1 hour each) before being impregnated with increasing concentrations of epoxy resin (TAAB medium grade; 25%, 50%, 75% in acetone, 3×100%, all for 1 hour each). They were then embedded in fresh resin and polymerized at 60°C for 24-36 hours. Sections were cut on an ultramicrotome, first semi-thin sections (0.5um) were stained with toluidine blue for LM to identify the area of interest/confirm orientation of tissue. Ultrathin sections (70nm) were then cut and picked up onto copper grids. Sections were stained with 1% uranyl acetate (30 min) and 3% lead citrate (7 min) on a Leica EM AC20 automatic staining machine before being viewed on the TEM (Philips CM100). Images were captured using a CCD camera (AMT40, Deben UK). Scanning for regions of interest ×5800 and initial images were captured at ×7900, more detailed images were captured at ×13500. Muscle fibers selected for analysis exhibited no more than two Z-lines separated by a minimum of 10-15 μm, ensuring optimal transverse orientation.

#### Serial block face scanning electron microscopy

Tissue was fixed in 2% glutaraldehyde in 0.1M cacodylate buffer. It was then processed using a heavy metal protocol adapted from (Wilke et al., 2013). Tissue was immersed in 3% potassium ferrocyanide + 2% osmium tetroxide (1 hour at 4°C), followed by filtered 0.1% thiocarbohydrazide (20 min), then 2% osmium tetroxide (30 min) and finally left overnight in 1% uranyl acetate at 4°C (with several water washes between each step). The next day the samples were immersed in 0.6% lead aspartate solution (30 min at 60°C and then dehydrated in graded acetone (as for TEM), impregnated with epoxy Taab 812 hard resin, embedded in fresh resin and polymerized at 60°C for 36-48 hours. After polymerization the block was sectioned for TEM to identify the area of interest then trimmed to approximately 0.75 mm by 0.5 mm and glued onto an aluminum pin. In order to reduce sample charging within the SEM, the block was painted with silver glue and sputter-coated with a 5 nm layer of gold. The pin was placed into a Zeiss Sigma SEM incorporating the Gatan 3view system for serial block face (SBF)-SEM, which allows sectioning of the block and the collection of serial images in the z-direction.

Using Digital Micrograph software, multiple regions of interest were selected and imaged at ×8235 magnification, 1024×1024 pixel scan, which gave a pixel resolution of approximately 10nm. Section thickness was 30nm in the z-direction and at least 400 sections were collected, yielding a total imaging depth of 12um. Before SBF-SEM, fibers with a high mitochondrial content were selected via TEM and a z stack then captured. For each human and mouse sample, four cells were imaged including threw for the IMF compartment, and one for the SS/perinuclear compartment.

#### 3D reconstructions

Image stacks from the SBF-SEM were converted to MRC files and 25 to 50 mitochondria reconstructed for each fiber using 3D-MOD (IMOD image analysis software, (IMOD 4.7, Boulder Laboratory for 3-D Electron Microscopy of Cells). Starting from the center of the stack to avoid mitochondria that would be interrupted at the edges of the images volume, randomly selected mitochondria were manually traced using the ‘sculpt’ drawing tool on each section. The OMM of each mitochondrion was followed in all three dimensions. Mitochondria with immediately adjacent OMM were segmented separately, and membrane continuity was established based on continuous planar electron density. A mesh and cap was then applied to fully segmented organelles using flat criteria value of 1.5 and all other parameters set as default, which enabled generation of closed volumes for surfaces lying between two 30nm slices in the Z plane. For each completely reconstructed mitochondrion, the total mesh surface area and total contour volume were extracted and used in analyses. SS mitochondria included in quantitative analyses were situated between myofibrils and the sarcolemma, often but not always in proximity to a nucleus, and did not protrude into the IMF space. To quantitatively distinguish between the two populations, SS mitochondria with IMF protrusions, as shown in Video S2, were not included in the analyses.

### QUANTIFICATION AND STATISTICAL ANALYSIS

#### Quantifying mitochondrial complexity

Mitochondrial complexity index (MCI) was calculated using the formula:

(1) MCI=(SA∕23)4πV

where SA is surface area and V is volume

This equation was derived from form factor (Koopman et al., 2005) and is a three-dimensional equivalent, used to assess mitochondrial morphological complexity. The fractional power is necessary to make the overall expression dimensionless (area dimensions of L^2^ has dimensions of L^3^), and thus independent of overall size of the object.

We tested this formula and confirmed that MCI is a measure of mitochondrial complexity invariant to volume (Figure S2). However, the scaling of MCI did not accurately represent the perceived increase in complexity. Changing the overall power of the complete expression has the effect of expanding the dynamic range of the measure without changing its dimensionality, and we investigated the effect of squaring and cubing the total formula. It was observed that squaring the measure gave the most accurate representation of mitochondrial complexity. As such the final formula employed was

(2) MCI=((SA∕23)4πV)2=SA316π2V2

Equations (1) and (2) contain the same information, but Equation (2) has a more natural mapping to perceived morphological difference.

MCI was compared to sphericity, another measure of three-dimensional shape that varies between 0 and 1, which asymptotically approaches 1 for a perfect sphere. MCI was selected over sphericity due to the theoretically unlimited complexity of mitochondrial morphology, which can scale up without an upper boundary as morphological complexity increases from sequential fusion events or increasing numbers of nanotunnels.

#### Quantifying mitochondrial branching

To assess mitochondrial branching and anisotropy, branching was quantified in the context of the surrounding tissue in the transverse and longitudinal planes and a ratio calculated. For transverse branching index the number of myofibrils bridged was counted and 1 added. The number of myofibrils bridged was calculated by adding together all branches that added to the distance covered in the transverse plane. If a branch curved or if the number of myofibrils was greater on one side than the other, the greatest value was taken (Figure S3A). For longitudinal branching the number of sarcomeres (+2), half sarcomeres (+1) and z-bands (+1) was summed and 1 added. Again only branches that added to the overall longitudinal distance were included (Figure S3B). The transverse branching score was divided by the longitudinal branching score, to yield a mitochondrial branching index (MBI). Mitochondrial were classified into 3 groups based on MBI; > 1 more branched in transverse orientation, 1 equally branched in both orientations and < 1 more branched longitudinally. Branching quantification was validated by comparison of assessments by two investigators, which yielded similar proportions of mitochondria established to be either more or equally branched in transverse and longitudinal orientations (Figure S3C-E). The inter-rater agreement in absolute MBI values was moderate (Figure S3C-E).

#### Nanotunnel measurements

For the purpose of mitochondrial nanotunnel measurements, previously identified mitochondria with nanotunnels were manually reconstructed in Microscopy Image Browser (Belevich et al., 2016) and exported as Amira mesh files for measurements analysis (Cocks et al., 2018). The total number of nanotunnels for which measurements were taken was 362, some nanotunnels were excluded from this analysis based morphology which was difficult to measure in Amira. The .mrc image stacks and Amira mesh models were opened in Amira to derive precise measurements of nanotunnel length, as well as minimum and maximum diameter along the length each nanotunnel.

#### Multivariate analysis and machine learning

We calculated summary statistics to describe the observed distributions of mitochondrial volume, complexity (MCI), and number of nanotunnels in cells from muscle biopsies of human patients, human controls and wild-type mice. Summary statistics included the percentage of mitochondria whose volume and MCI were in the 10^th^ percentile (bottom 10% of the distribution) or in the 90^th^ percentile (top 10%) of the combined control population (percent large, complex, percent small, and simple, respectively) as well as the number of nanotunnels per 100 mitochondria and mitochondrial volume density. Partial least-squares discriminant analysis (PLS-DA) was performed using the mixOmics (6.3.1) (Lê Cao et al., 2009; Rohart et al., 2017) R (3.4.3) (Team, 2017) package (code available: https://gist.github.com/CnrLwlss/e4b0b2a2c4290ae4ad4eb9a4373680f3). Variable importance in projection (VIP) scores were extracted for each mitochondrial feature from PLS-DA models.

#### Statistical analyses

Data on volume and MCI were fit using a linear mixed model with organism and cell (nested within organism) as random effects and species/disease status as fixed effects. Because of the extent of the skewness of the data, measurements were log-transformed before analysis. For all other statistics, normalization was not effective and therefore non-parametric Mann-Whitney tests were used. For branching analyses a chi square test was used to determine the significance for the percentages of mitochondria classified in each group.

### DATA AND SOFTWARE AVAILABILITY

Image stacks and model files are available from the authors upon request.

## Supplementary Material

1

2

3

4

5

6

7

8

## Figures and Tables

**Table:** KEY RESOURCES TABLE

REAGENT or RESOURCE	SOURCE	IDENTIFIER
Chemicals, Peptides and Recombinant Proteins		
Acetone	Fisher	A060617
Aluminum pin	Taab	G312
Aspartic acid	Sigma Aldrich	A9256
Cacodylate buffer	Agar Scientific	R1104
Copper grids	Gilder grids	GA 1500-C3
Epoxy resin	Taab	T030
Glutaraldehyde	Taab	G003
Gold coating	Agar Scientific	B7370
Lead Nitrate	Sigma-Aldrich	228621
potassium ferrocyanide	Sigma-Aldrich	244023
osmium tetroxide	Agar scientific	AGR1024
thiocarbohydrazide	Sigma-Aldrich	223220
Toluidine blue	Taab	SD211
Silver glue	Agar scientific	G3648
Uranyl acetate	Agar scientific	AGR1260A

Experimental Models:Organisms/Strains		
PV-Cre Knockin mice on c57bl/6N background (male)	Jackson lab	008069
Pentobarbital containing Euthatal	Henry Schein	MBEUT01

Software and Algorithms		
3DMOD/IMOD	Boulder Lab, University of Colorado	https://bio3d.colorado.edu/imod/; RRID:SCR_003297
Amira	Thermo Fisher Scientifc	https://www.fei.com/software/amira-3d-for-life-sciences/; RRID:SCR_007353
Digital Micrograph	Gatan	http://www.gatan.com/products/tem-analysis/gatan-microscopy-suite-software
MixOmics (6.3.1)		https://cran.r-project.org/web/packages/mixOmics/index.html
Prism 7		https://www.graphpad.com/scientific-software/prism/
R (3.4.3)		https://www.r-project.org/
Multi-variate statistical analysis code	This paper	https://doi.org/10.5281/zenodo.2528488

Other		
Serial block face SEM.	Gatan	3view
SEM.	Zeiss	Sigma
TEM	Phillips	CM100
CCD camera	Deben	AMT40

## Highlights

- 3D mitochondrial morphology is quantified by the mitochondrial complexity index (MCI)
- Mouse mitochondria are larger and exhibit greater connectivity than human mitochondria
- Non-adjacent mitochondria are connected by narrow mitochondrial nanotunnels
- Multivariate morphology signatures distinguish mitochondrial disease patients from healthy controls
