# Quantitative 3D Mapping of the Human Skeletal Muscle Mitochondrial Network

**DOI:** 10.1016/j.celrep.2019.03.051

**Published:** 2019-04-02

**Authors:** Amy E. Vincent, Kathryn White, Tracey Davey, Jonathan Philips, R. Todd Ogden, Conor Lawless, Charlotte Warren, Matt G. Hall, Yi Shiau Ng, Gavin Falkous, Thomas Holden, David Deehan, Robert W. Taylor, Doug M. Turnbull, Martin Picard

In the version of this article originally published online, there was a misspelling of the name Conor Lawless in the author list. This has now been corrected online. The authors apologize for this error.

