# Supplementary material for: Quantitative 3D Mapping of the Human Skeletal Muscle Mitochondrial Network
Source: Cell Rep. Author manuscript; Available in PMC 2019 May 13. (PMC6513570; doi:10.1016/j.celrep.2019.01.010)
Supplement: 1 [file NIHMS1519374-supplement-1.pdf]

Cell Reports, Volume *26*

Supplemental Information

Quantitative 3D Mapping of the Human Skeletal Muscle Mitochondrial Network

Amy E. Vincent, Kathryn White, Tracey Davey, Jonathan Philips, R. Todd Ogden, Conor Lawess, Charlotte Warren, Matt G. Hall, Yi Shiau Ng, Gavin Falkous, Thomas Holden, David Deehan, Robert W. Taylor, Doug M. Turnbull, and Martin Picard

Table S1. Clinical and diagnostic information for patients with mitochondrial disease. Related to STAR methods.

Patient number	Sex	Age at biopsy	Mitochondrial genetic diagnosis	Heteroplasmy	% COX deficiency	% RRF	Clinical phenotype	Notes
**1**	F	62	single, large-scale, mtDNA deletion	34	20	15	CPEO, proximal myopathy	None
**2**	F	70	single, large-scale, mtDNA deletion	22	18	9	CPEO and myopathy	None
**3**	F	22	m.8344A>G	97	97	10	MERRF	Severly affected – myopathy
**4**	F	50	m.8344A>G	63	22	7	Mild myopathy	Mother of patient 3
**5**	F	20	m.8344A>G	40	Some COX-intermediate fibres	0	Asymptomatic	Sister of patient 3
**6**	F	69	m.3243A>G	21	3	2	MIDD	None

% COX deficiency: proportion of myofibers with cytochrome c oxidase deficiency; RRF: ragged red fibers; CPEO: chronic progressive external opthalmoplegia; MERRF: myoclonic epilepsy with ragged red fibers; MIDD: Maternally Inherited Deafness and Diabetes. Deletion position and sizes for Patient 1: m.8482-13460, 4977bp; Patient 2: m.8576-12968, 4391bp. All patient muscles sampled were taken from tibialis anterior muscle.

Table S2. Sex and age for healthy control skeletal muscle biopsies from routine anterior cruciate ligament (ACL) surgery. Related to STAR methods.

Control ID	Sex	Age at biopsy
**1**	M	23
**2**	F	25
**3**	M	25
**4**	M	27
**5**	M	28
**6**	M	33
**7**	F	35
**8**	F	45

Supplemental Figure 1

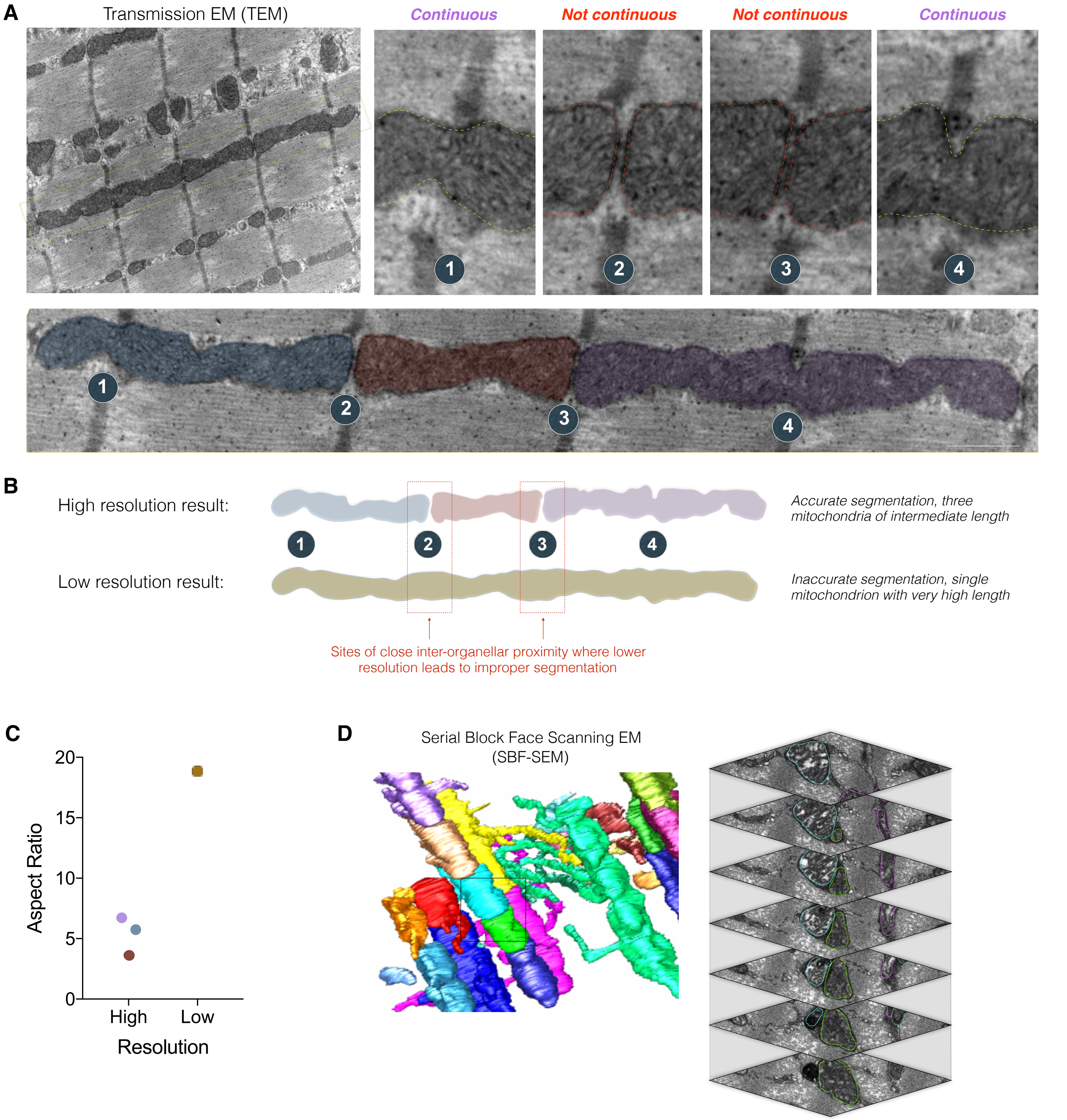

Figure S1. Method validation by comparison with mice. Related to Figure 1.

(**A**) Transmission electron micrograph (TEM) of mouse skeletal muscle with columnar inter-myofibrillar (IMF) mitochondria that could mistakenly be segmented as a single mitochondrion from images with insufficient resolution.

(**B**) Results of segmentation of mitochondria in (A) with either high and low resolution imaging.

(**C**) Quantification of aspect ratio for mitochondria in (A) and (B). Low resolution imaging leads to overestimation of mitochondrial size, length, and branching, and underestimation of mitochondrial numbers. Points are coloured to match mitochondria in (A) and (B)

(**D**) Example reconstruction from high-resolution serial block-face scanning electron microscopy in mouse (*left*) and image stack with adjacent mitochondria appropriately segmented (*right*).

Supplemental Figure 2

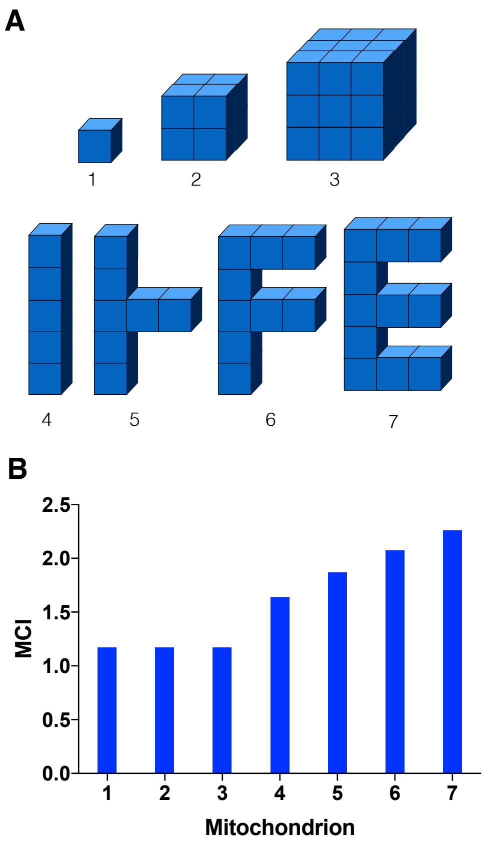

Figure S2. Validation of the mitochondrial complexity index (MCI) as a shape descriptor. Related to Figure 2-5.
(**A**) Three-dimensional shapes (mitochondria) constructed from 1x1x1 blocks for which volume and surface area can be determined.
(**B**) Calculated MCI values for shapes in (A). This analysis confirms that i) MCI is insensitive to volume, as indicated in the absence of change in MCI values for the cubes of increasing volumes (Objects 1-3); and ii) MCI is a three-dimensional measure of morphological complexity, scaling quasi-linearly with the number of branches (Objects 4-7).

Supplemental Figure 3

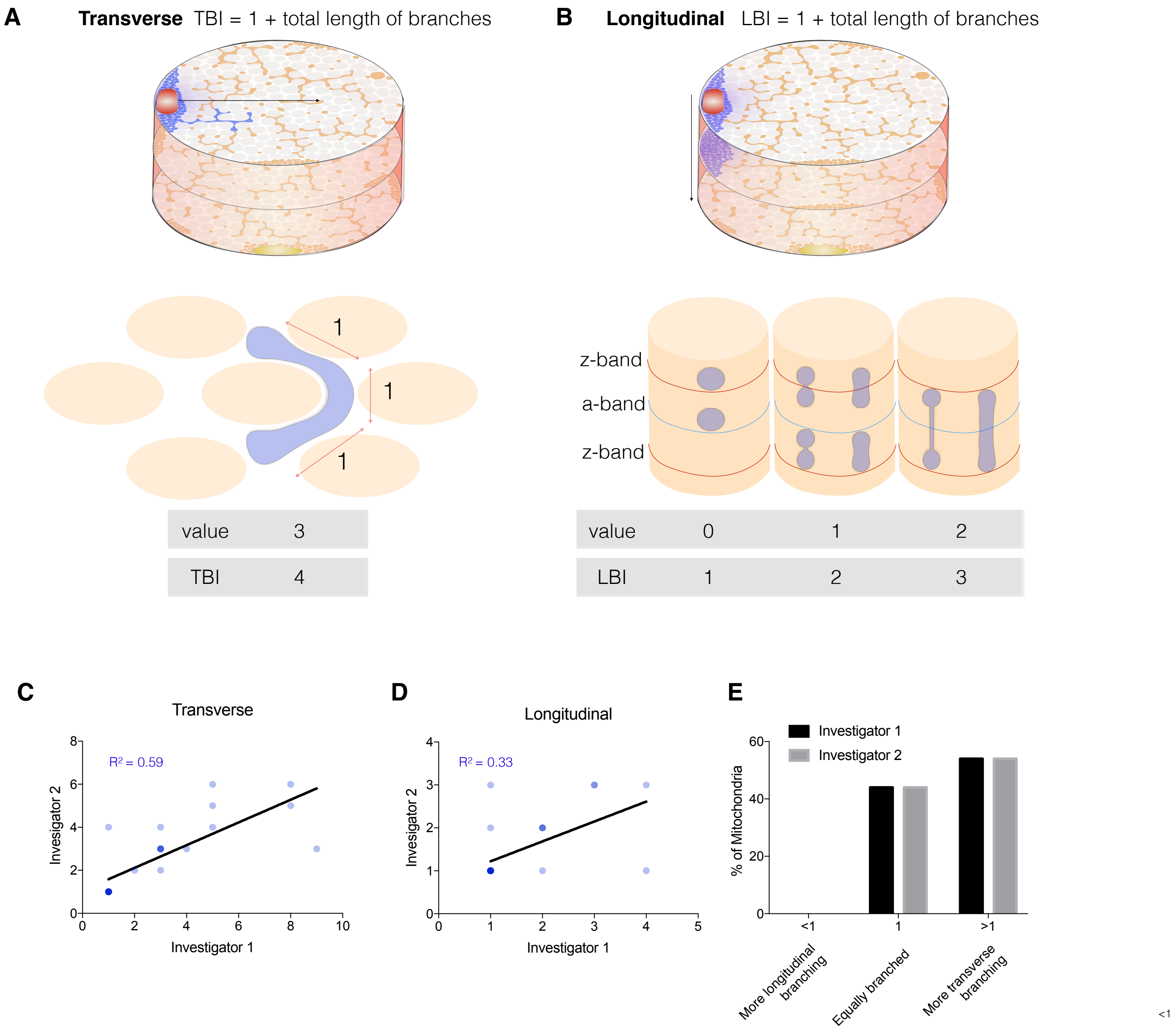

Figure S3. Quantification of transverse and longitudinal mitochondrial branching to assess anisotropy. Related to Figures 1 and 4.

(**A**) Branching in cross-section is assessed as CBI = 1 + the total length of all branches. The total length of branches is the sum of each myofibril "bridged". In the event that a mitochondrion wraps around one myofibril as depicted the side with the greatest value is taken (i.e. this mitochondrion bridges three myofibrils.

(**B**) Branching in longitudinal orientation is assessed as LBI = 1 + total longitudinal boundaries bridged. Bridging of a z-band or half a sarcomere is valued as one, bridging of a full sarcomere is valued as 2.

(**C**) Inter-rater variability of transverse and (**D**) longitudinal branching quantification as assessed by comparison of investigators 1 and 2 in assessment of mitochondria (n=25 per orientation).

(**E**) Classification of mitochondria based on mitochondrial branching index (MBI). MBI = TBI/LBI. Mitochondria are classified as; more extensively branched in the transverse orientation (MBI Score >1), equally branched in both directions (MBI = 1) or more branched longitudinally (MBI < 1).

Supplemental Figure 4

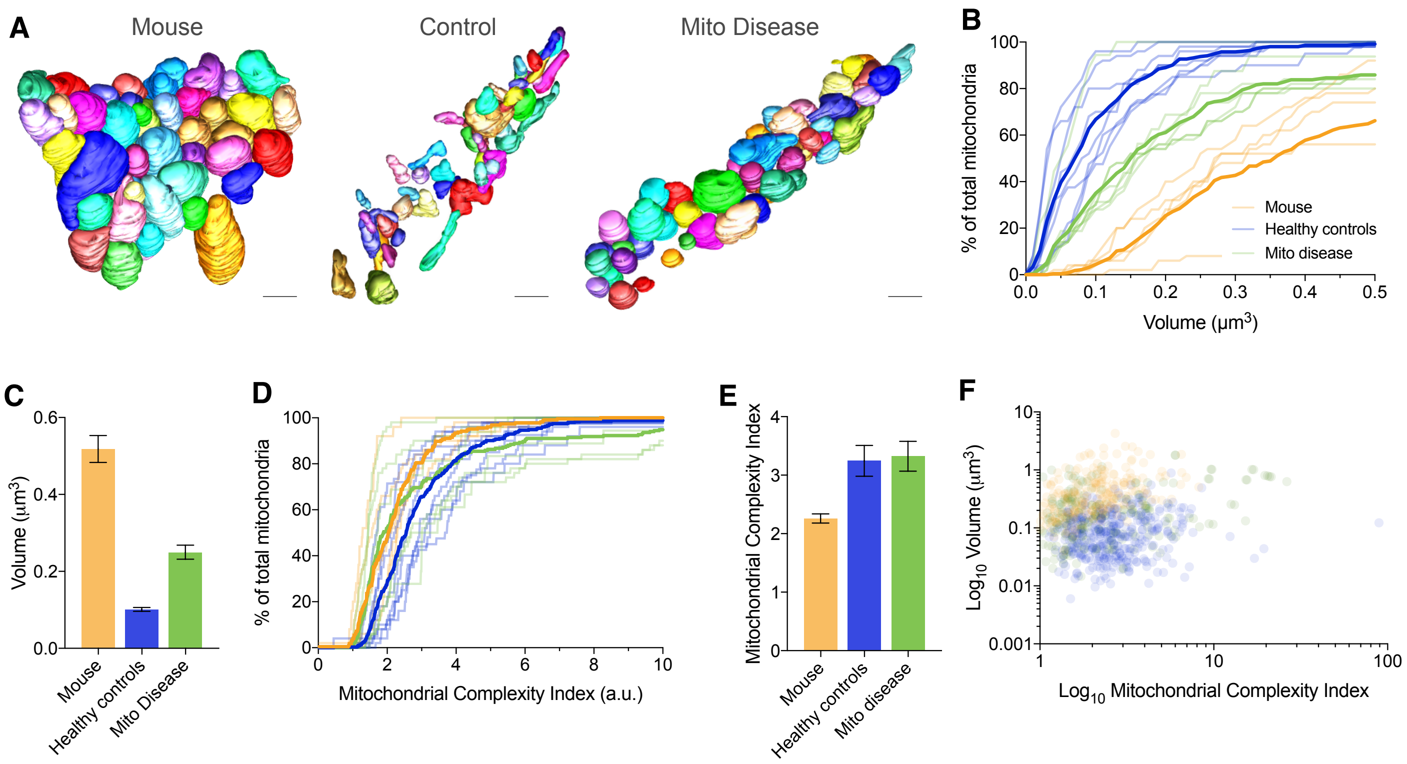

Figure S4. MCI and volume for subsarcolemmal mitochondria in mice, controls and patients. Related to Figures 1 and 3.

(**A**) Example three dimensional reconstruction of subsarcolemmal mitochondria (n = 50) from mouse 2, healthy control sample 2 and mitochondrial disease patient 3 (m.8344A>G at 97% mutation load). Each mitochondrion is coloured differently.

(**B**) Cumulative frequency distribution for mitochondrial volume in individual mice (*orange*), healthy controls (*blue*) and mitochondrial disease (*green*).

(**C**) Bar chart showing mean and SEM for mitochondrial complexity index in mice, control humans and mitochondrial disease patients.

(**D**) Cumulative frequency distribution for mitochondrial mitochondrial complexity index in individual mice (*orange*), healthy controls (*blue*) and mitochondrial disease (*green*).

(**E**) Bar chart showing mean and SEM for mitochondrial complexity index in mice, healthy humans and mitochondrial disease.

(**F**) Log10 volume plotted against Log10 mitochondrial complexity index for each subsarcolemmal mitochondrion from combined mice (*orange*), healthy controls (*blue*) and mitochondrial disease (green).

Scale bars = 1μm

Supplemental Figure 5

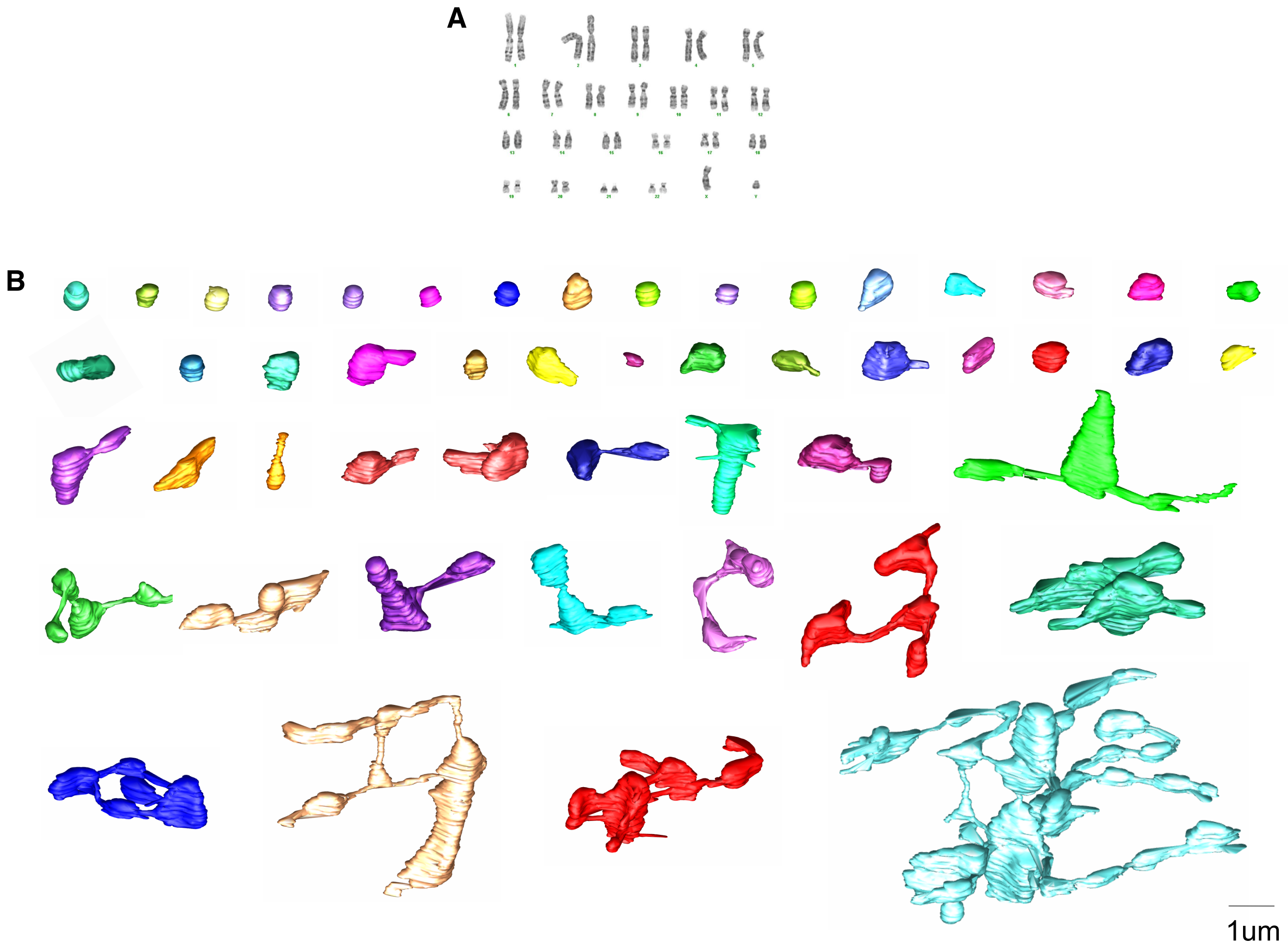

Figure S5. Mito-otype of a human muscle fiber. Related to Figure 3.
(**A**) Standard karyotype for a eukaryotic cell's chromosomes organized by appearance.
(**B**) Equivalent organization of 50 individual reconstructed IMF mitochondria from a human muscle fiber organized by complexity, from the lowest to the highest MCI (*left to right*). This mito-otype was generated from the reconstructed muscle fiber animated in Video S3, where each mitochondrion can be seen in the context of other mitochondria.

Supplemental Figure 6

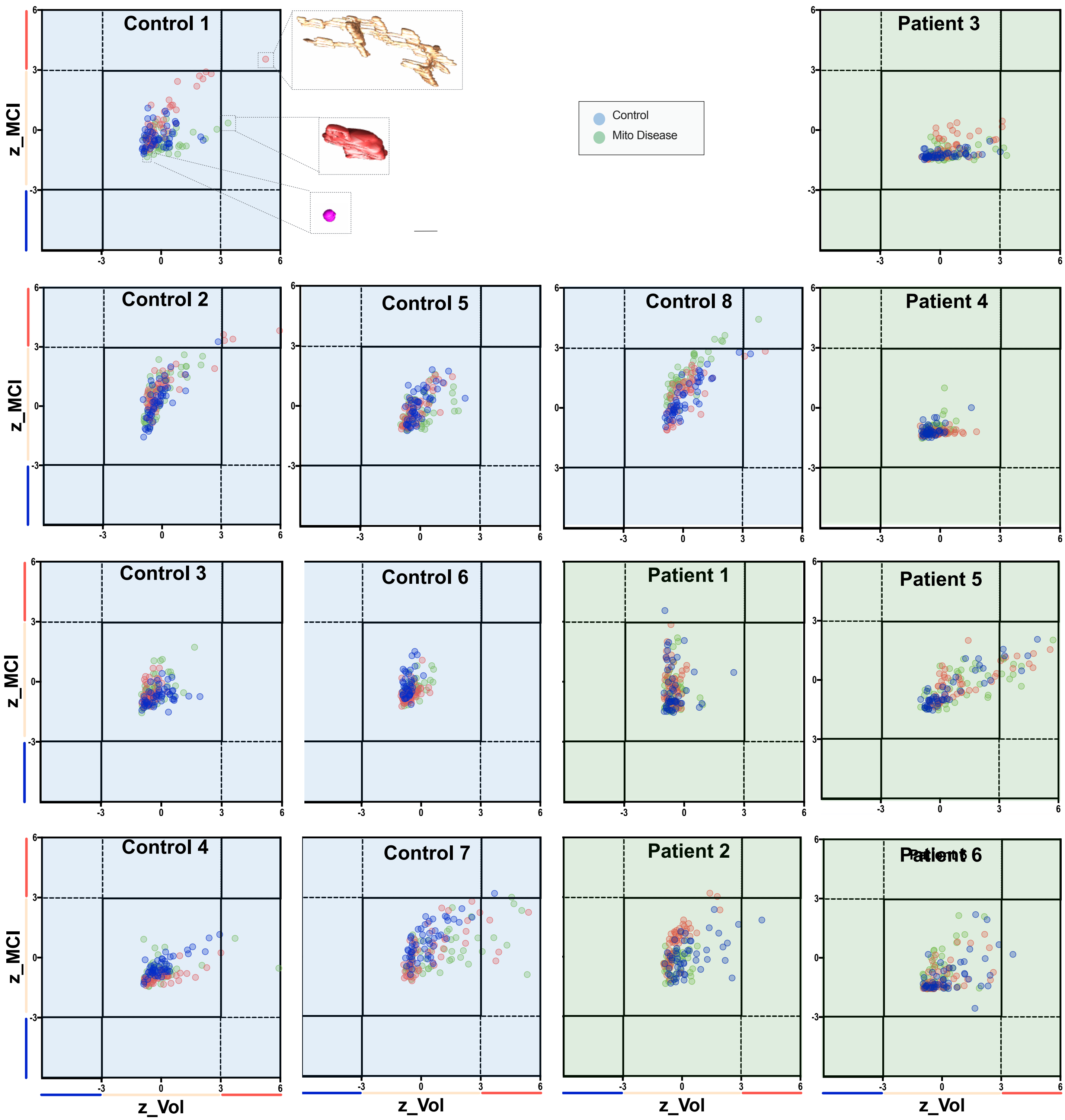

Figure S6. MCI and volume of controls and patients. Related to Figures 3 and 4.
The z-score for MCI was plotted against the z-score for volume of each mitochondrion. The z-score is the deviation from the mean of the healthy control population. Each fibre is plotted in a different colour. These graphs demonstrate that the controls generally have a wider range of MCI and volume. In comparison the mitochondrial disease cases have one of three patterns of interest. Patients 3 and 4 have a small range of MCI and volume for all fibres. Whereas for patient 2 one fibre (*green*) has a smaller range than the other two (*red and blue*). For patients 1, 5 and 6 all fibres appear to be equally spread.
